# Gegen Qinlian Decoction, a classic traditional Chinese medicine formula: a potential therapeutic strategy for ulcerative colitis and colorectal cancer

**DOI:** 10.3389/fphar.2026.1868875

**Published:** 2026-07-15

**Authors:** Yijun Hou, Han Wang, Jitong Li, Chang Li, Yanting Sun, Yurui Wang, Dixin Zou, Liya Zhou

**Affiliations:** 1 College of Traditional Chinese Medicine, Changchun University of Chinese Medicine, Changchun, China; 2 Department of Hepatology, Splenology and Gastroenterology, The First Affiliated Hospital of Changchun University of Traditional Chinese Medicine, Changchun, China; 3 Basic Medical College, Changchun University of Chinese Medicine, Changchun, China; 4 State Key Laboratory for Quality Ensurance and Sustainable Use of Dao-di Herbs, Institute of Chinese Materia Medica, China Academy of Chinese Medical Sciences, Beijing, China

**Keywords:** colitis-associated colorectal cancer, colorectal cancer, Gegen Qinlian Decoction, gut microbiota, Inflammation–carcinogenesis transition, intestinal barrier, traditional Chinese medicine, ulcerative colitis

## Abstract

Gegen Qinlian Decoction (GQD), a classic traditional Chinese medicine formula recorded in Shang Han Lun, has been used clinically for approximately two thousand years. In recent years, its potential roles in ulcerative colitis (UC) and colorectal cancer (CRC) have attracted increasing attention because of the multi-component, multi-target, and multi-pathway regulatory characteristics of traditional Chinese medicine (TCM). UC is a chronic relapsing inflammatory disease and an important risk factor for CRC. Although emerging immunomodulators, biologics, and targeted therapies have achieved certain efficacy in the treatment of UC and CRC, limited response, drug resistance, adverse effects, and disease recurrence remain major challenges for some patients. Persistent intestinal inflammation can disrupt the epithelial barrier, alter gut microbiota, induce immune imbalance, enhance oxidative stress, and activate pro-tumorigenic signaling, thereby contributing to the transition from UC to CRC. Within this inflammation-to-cancer framework, preclinical evidence suggests that GQD may exert comprehensive regulatory effects on multiple pathological processes. It may suppress inflammatory signaling pathways such as TLR4/NF-κB, IL-6/JAK2/STAT3, and the NLRP3 inflammasome, regulate Th17/Treg balance and macrophage polarization, restore epithelial barrier integrity, reshape gut microbiota homeostasis, and modulate oxidative stress, abnormal cell death, and metabolic dysregulation. These effects may act synergistically to limit the transition from an inflammatory microenvironment to a pro-tumorigenic microenvironment. In established CRC, GQD appears more suitable as a potential adjunctive therapy rather than a primary cytotoxic anticancer agent, with possible roles in reducing inflammation-associated tumor-promoting signals, remodeling the tumor immune microenvironment, enhancing responses to chemotherapy or immunotherapy, and mitigating treatment-related intestinal toxicity. In summary, GQD may have potential important value in the treatment of UC and CRC, and this review provides a reference for future research and clinical application.

## 1 Introduction

Ulcerative colitis (UC) is a chronic, relapsing inflammatory disorder of the colon, mainly characterized by recurrent diarrhea, abdominal pain, and mucopurulent bloody stool, and it poses a substantial burden on global health (Lee et al., 2018). Epidemiological studies have reported approximately 5 million UC cases in 2023 globally, and the incidence is increasing annually. The incidence of inflammatory bowel disease is highest in China among Asian countries and exhibits a significant upward trend; the incidence of UC in China is 11.6 cases per 100,000 person-years (Wang et al., 2013; Yu Q. et al., 2021; Le Berre et al., 2023). UC is diagnosed based on a combination of clinical, endoscopic, and histopathological findings. Endoscopically, mucosal inflammation and ulceration are key features, typically beginning in the rectum and extending proximally to the colon or even the entire colon (pancolitis) (Ungaro et al., 2017; Buie et al., 2023; Le Berre et al., 2023; Liang Y. et al., 2024). Conventional UC therapies include anti-inflammatory pharmacotherapy, such as aminosalicylates, corticosteroids, and immunosuppressants; symptomatic management, including water–electrolyte regulation and antibiotics; and surgical resection. Novel agents, including biologics and targeted therapies, have reduced morbidity, mortality, and colectomy rates, but limitations remain, including single-mechanism dependence, drug resistance, and risks of infection and malignancy (Magro et al., 2014; Burri et al., 2020; Dai et al., 2023; Wang K. et al., 2024). However, the risk of colorectal cancer (CRC) is 2- to 3-fold higher in patients with chronic and recurrent UC compared with the general population, accounting for 15% of all-cause mortality in this cohort. CRC represents the most severe clinical complication and is the leading cause of mortality and a primary indication for colectomy in this patient population (Nagao-Kitamoto et al., 2022; Shah and Itzkowitz, 2022; Xu Y. et al., 2024; Sun R. et al., 2025). The application of chemotherapeutic agents, radiotherapy, and various emerging targeted therapies—such as monoclonal antibodies—has offered new promising therapeutic avenues for patients with CRC. However, clinical benefits are frequently transient due to intrinsic and acquired drug resistance mechanisms, as well as complex pathophysiological factors (Li Q. et al., 2024). These limitations highlight the need for alternative or complementary therapeutic strategies capable of intervening in multiple pathological processes simultaneously.

Traditional Chinese medicine (TCM) has been widely used in the management of UC and CRC and is characterized by multi-component, multi-target, and multi-pathway modes of action. Within this context, Gegen Qinlian Decoction (GQD) is a classical and frequently used herbal formula. First recorded in the Shanghan Lun (Treatise on Febrile Diseases) by the renowned physician Zhang Zhongjing during the Han Dynasty, GQD consists of four medicinal botanical drugs: *Puerariae Lobatae Radix* (Gegen), *Scutellariae Radix* (Huangqin), *Coptidis Rhizoma* (Huanglian),*Glycyrrhizae Radix et Rhizoma* (Gancao). All botanical drugs included in GQD were taxonomically validated using Plants of the World Online (POWO, http://www.plantsoftheworldonline.org), and the full scientific names, authorities, and families are provided in Table 1. Traditionally, GQD has been used to clear heat, resolve dampness, and treat diarrhea-related disorders associated with damp-heat syndrome. Modern studies have further indicated that GQD has therapeutic potential in a variety of diseases, including UC (Xu et al., 2015; Li et al., 2016; Zhao et al., 2020), diabetes mellitus (Tu et al., 2020; Tan et al., 2024), non-alcoholic fatty liver disease (NAFLD) (Guo et al., 2018; Zhang et al., 2020), and tumors (Wang et al., 2015; Lv et al., 2019). Previous studies indicate that GQD potentially mitigates UC via multiple pathways, including alleviation of intestinal inflammation and oxidative stress (Wang Y. et al., 2023), immunomodulation (Ma et al., 2023), and regulation of gut microbiota and metabolites (Huang J. et al., 2024). GQD treatment reduces recurrence rates and mitigates adverse drug reactions (Fan et al., 2019; Chen Y. et al., 2025). With respect to CRC, however, the currently available evidence does not support GQD as a standalone anticancer treatment. Rather, existing experimental findings and limited clinical observations suggest that it may play a supportive or adjunctive role, for example by synergizing with modern pharmacotherapies and helping to mitigate drug resistance (Fan et al., 2019; Lin et al., 2024; Xu Y. et al., 2024; Chen Y. et al., 2025).

**TABLE 1:** The botanical drugs composition of GQD.

Traditional name (Pinyin)	Pharmaceutical name	Plant part used	Accepted botanical name and authority (Validated via POWO)	Family
Gegen	Puerariae lobatae radix	Root (Radix)	Pueraria montana var. lobata (willd.) maesen & S.M.Almeida ex sanjappa & predeep	Fabaceae
Huangqin	Scutellariae radix	Root (Radix)	Scutellaria baicalensis georgi	Lamiaceae
Huanglian	Coptidis rhizoma	Rhizome (Rhizoma)	Coptis chinensis franch	Ranunculaceae
Gancao	Glycyrrhizae radix et rhizoma	Root and rhizome (Radix et Rhizoma)	Glycyrrhiza uralensis Fisch. ex DC.	Fabaceae

Previous reviews have mainly summarized the clinical efficacy and pharmacological effects of GQD in specific disease contexts, such as UC, metabolic diseases, and tumors, involving anti-inflammatory effects, regulation of gut microbiota, and antitumor activity. However, systematic discussions on the potential role of GQD in the UC–CRC inflammation-to-cancer transition remain relatively limited. This review focuses on the continuous process of UC progression to CRC and systematically summarizes how GQD may affect UC by regulating multiple pathological nodes, including immune imbalance, gut microbiota dysbiosis, epithelial barrier injury, oxidative stress, abnormal cell death, and metabolic dysregulation. Meanwhile, this review cautiously positions GQD as a potential adjunctive therapeutic strategy, with particular attention to its potential value in reshaping gut microbiota, modulating the tumor immune microenvironment, improving treatment tolerance, regulating drug resistance, and other related aspects. In addition, this review proposes strategies that may contribute to further improvement from multiple aspects, including formula standardization and bioavailability optimization, with the aim of providing scientific evidence and references for basic research and current clinical applications.

## 2 From UC to CRC: pathological continuum and key mechanistic basis

**FIGURE 1:**
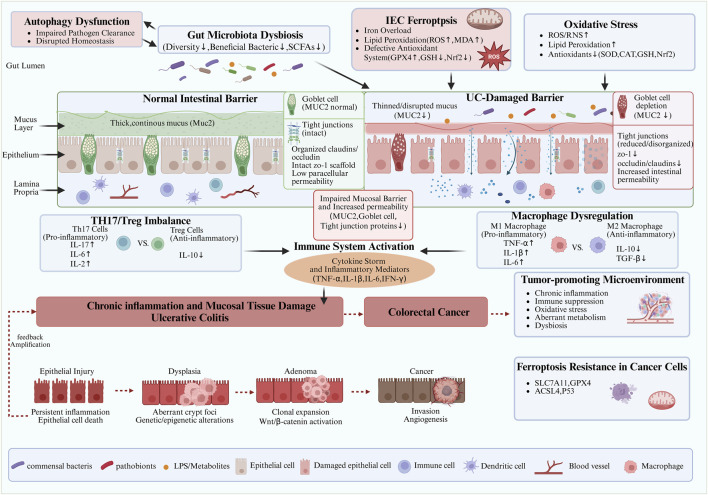
Schematic illustration of the key mechanisms underlying the pathological continuum from UC to CRC.

Since the mid-20th century, UC incidence has steadily risen worldwide, posing a significant public health burden. UC is marked by intestinal mucosal barrier disruption, leading to unresolved mucosal injury (Mak et al., 2020; Saez et al., 2023). CRC, a major long-term complication of UC, is the third most commonly diagnosed malignancy and the second leading cause of cancer-related mortality, with cases projected to reach over 2.2 million and 1.1 million deaths by 2030 (Arnold et al., 2017; Bray et al., 2024). Chronic intestinal inflammation, epithelial barrier disruption, oxidative stress, and microenvironmental remodeling collectively drive the transition from mucosal injury to carcinogenesis. Increasing evidence indicates that persistent inflammatory burden not only sustains disease activity in UC but also promotes tumor initiation and progression, particularly in colitis-associated colorectal cancer (CAC). The underlying mechanisms are summarized below and illustrated in Figure 1.

### 2.1 Chronic intestinal inflammation and immune dysregulation

Aberrant immune activation and persistent inflammatory responses are central to the pathogenesis of UC and also constitute a key mechanistic link to CRC development. The potential pathophysiological link between defective immune function and chronic intestinal inflammation has been reported in several studies. Studies have indicated that patients with primary immunodeficiencies (PID) have an approximately 80-fold higher risk of developing inflammatory bowel disease (IBD) compared with the general population (Fischer et al., 2017), and PID is present in about 20% of very early-onset inflammatory bowel disease (VEO-IBD) cases (Ouahed et al., 2020). A significant association may exist between human immunodeficiency virus (HIV) infection and the development of IBD. However, because of the complexity in the underlying mechanisms, the precise relationship remains a subject of considerable debate within the academic community. Viazis et al. reported that HIV infection exerts a protective effect in patients with IBD, thereby suggesting a potential role for the CD4^+^ T cells (Viazis et al., 2010). Conversely, other studies suggest that patients with HIV infection are at a significantly increased risk of developing IBD (Huang W. et al., 2024). Therefore, further investigations are necessary to elucidate definitive relationship between these two conditions. Immunodeficiency is also closely associated with an increased risk of malignancy, and the underlying mechanisms mainly include impaired immune surveillance, persistent or recurrent infections, genomic instability caused by defective DNA repair, and abnormalities in specific immune cell and cytokine pathways that collectively promote tumorigenesis (Mortaz et al., 2016). The relationship between immunodeficiency and tumorigenesis is complex. Infections related to immune defects may drive chronic inflammation and aberrant tissue repair, while impaired immune surveillance can further promote malignancy. Although evidence is insufficient to confirm that HIV increases CRC risk, HIV-induced colorectal inflammation and barrier disruption may foster a pro-tumorigenic microenvironment (OʼNeill et al., 2017; Rocha et al., 2025).

Gut microbiota dysbiosis and intestinal barrier impairment increase intestinal permeability, thereby activating mucosal immune responses and inducing local secretion of pro-inflammatory cytokines, including interleukin-1β (IL-1β), interleukin-6 (IL-6), tumor necrosis factor-α (TNF-α), and interferon-γ (IFN-γ), ultimately contributing to UC development (Kobayashi et al., 2020). The disruption of equilibrium between helper T (Th) cells and regulatory T cells (Tregs) represents a fundamental mechanism underlying IBD. In contrast to Crohn’s disease (CD), which is primarily driven by a Th1 response, UC is predominantly mediated by Th2 cell-derived cytokines, including interleukin-4 (IL-4), interleukin-5 (IL-5), interleukin-10 (IL-10), and interleukin-13 (IL-13) (Muro et al., 2024). T lymphocytes are pivotal immune cells. When activated, they release a variety of cytokines and vasoactive substances, thereby exacerbating inflammatory responses. As a subset of T cells, CD4^+^ T cells differentiate into T helper 17 (Th17) cells and Tregs. The equilibrium between these 2 cell populations is critical for maintaining inflammatory homeostasis. Hyperactivated Th17 cells exacerbate UC by secreting pro-inflammatory cytokines such as interleukin-2 (IL-2), IL-6, and interleukin-17 (IL-17), whereas Tregs suppress immune-mediated inflammation partly through IL-10. However, Treg function is often impaired in UC, further amplifying inflammatory responses (Campbell et al., 2018; Cronkite and Strutt, 2018; Kobayashi et al., 2020; Yan Y. et al., 2023; Zhao et al., 2023). Therefore, restoring the Th17/Treg balance and modulating pro-inflammatory cytokines may represent potential therapeutic strategies for UC (Zhu et al., 2025).

Importantly, the persistent inflammatory milieu established in UC also provides a mechanistic basis for colorectal tumorigenesis. The ‘inflammation-carcinogenesis’ pathway has been well substantiated. Prolonged chronic inflammation fosters an environment conducive to cancer cell development, inducing angiogenesis, fibrosis, tissue destruction, and cancer progression. The persistence of inflammatory cells and inflammatory factors within the tumor microenvironment (TME) accelerates tumor growth and inhibits apoptosis of transformed cells (Korniluk et al., 2017; Sohrab et al., 2023). In patients with IBD, the increased risk of developing CRC, especially CAC, strongly correlates with the cumulative inflammatory burden (Yvellez et al., 2021). Inflammatory mediators released from inflamed tissues can facilitate all stages of tumorigenesis by promoting tumor initiation (inducing DNA damage involving oncogenes and/or tumor suppressor genes) and by enhancing the proliferation and resistance to apoptosis of mutated cells. For example, IL-6, IL-1β, TNF-α, IL-10, and nitric oxide (NO) promotes transition from chronic intestinal inflammation to cancer (Kirchberger et al., 2013; Brighenti et al., 2014; Chen et al., 2021a). Furthermore, macrophages are innate immune cells that are involved in the pathogenesis of UC and the transition from UC to CRC.

Macrophages constitute a crucial cellular component of innate immune defense and are commonly classified into two functional activation states: classically activated M1 macrophages and alternatively activated M2 macrophages. In early UC, M1 macrophages predominate, secreting pro-inflammatory cytokines such as TNF-α, IL-1β, and IL-6, which recruit and activate other immune cells, forming a self-sustaining inflammatory loop (Zhang Q. et al., 2022). During chronic or resolution phases, M2 macrophages suppress inflammation and support mucosal healing via anti-inflammatory mediators, including transforming growth factor-β (TGF-β) and IL-10, and contribute to extracellular matrix remodeling, tissue regeneration, and angiogenesis. Disruption of M1/M2 balance is closely linked to persistent intestinal inflammation in UC, and restoring this balance may represent a therapeutic strategy (Yang et al., 2022). In CRC, especially CAC, macrophages have context-dependent dual roles. Sustained macrophage infiltration during inflammation-driven tumorigenesis can promote neoplastic progression via pro-inflammatory cytokines and activation of IL-6/Signal Transducer and Activator of Transcription 3 (STAT3) and TNF-α/NF-κB pathways. After tumor formation, M1 macrophages generally exert anti-tumor effects through enhanced antigen presentation and cytotoxicity of CD8^+^ T and NK cells, whereas M2 macrophages support tumor progression by promoting angiogenesis, suppressing immunity, and facilitating invasion and metastasis (Zhang et al., 2023). Thus, macrophages not only contribute to inflammation-associated colorectal tumorigenesis, but also actively shape the tumor microenvironment after malignant transformation.

### 2.2 Gut microbiota dysbiosis and intestinal barrier disruption

The intestine harbors a diverse microbial community that interacts with the host to maintain intestinal microecological balance and mucosal barrier integrity. Under pathological conditions, alterations in gut microbiota composition and abundance disrupt the intestinal barrier, promote translocation of endotoxins and harmful substances, and induce intestinal injury. Gut microbiota from patients with IBD can influence the Th17/Treg balance in the murine intestine and trigger intestinal immune inflammation (Britton et al., 2019). Gut microbiota dysbiosis is an important contributor to UC (Guo et al., 2020), typically characterized by reduced microbial diversity and depletion of beneficial bacteria such as *Faecalibacterium prausnitzii*, *Bifidobacterium* spp., and *Clostridium coccoides* (Poppe et al., 2024). In the gut microbiota of mice with UC, reduced abundance of *Firmicutes* and increased abundance of *Bacteroidetes* reduces the *Firmicutes/Bacteroidetes* (F/B) ratio, which is closely associated with the pathogenesis of UC (Shen et al., 2018; Stojanov et al., 2020; Zou et al., 2023). Dysbiosis also increases pathogenic bacteria and lipopolysaccharide (LPS) levels, thereby activating Toll-like receptor 4 (TLR4) signaling, promoting pro-inflammatory cytokine release, and further impairing the intestinal barrier (Xiong et al., 2022). Beyond UC, persistent gut dysbiosis contributes to CRC initiation and progression, characterized by enrichment of pro-carcinogenic bacteria, including *Fusobacterium nucleatum, enterotoxigenic Bacteroides fragilis* (ETBF), and *pks + Escherichia coli*, together with depletion of protective commensals and short-chain fatty acid-producing bacteria. These microbial alterations may promote colorectal tumorigenesis through barrier disruption, chronic inflammatory signaling, genotoxic stress, and oncogenic pathway activation (Sheflin et al., 2014; Garvey, 2024; Kang et al., 2025).

Gut microbiota-derived metabolites—especially short-chain fatty acids (SCFAs), bile acids, and tryptophan (Trp)—play a critical role in regulating microbial composition and maintaining intestinal barrier integrity and immune homeostasis (Liu et al., 2022). SCFAs are metabolites derived from the fermentation of dietary fiber by the gut microbiota. They regulate differentiation of CD4^+^ T cells, restore balance of the immune axis, and inhibit inflammatory responses. SCFAs primarily consist of acetate, propionate, and butyrate. Among these, butyrate acts as an agonist for peroxisome proliferator-activated receptor-γ (PPARγ), a member of the nuclear receptor superfamily that plays a pivotal role in maintaining intestinal homeostasis. Butyrate reduces intestinal permeability, enhances the intestinal mucosal barrier, downregulates the expression of the pro-inflammatory cytokine IL-6, and activates PPARγ, thereby ameliorating the severity of UC (Park et al., 2015; Holscher, 2017; Venkataraman et al., 2020; Wang et al., 2022). In CRC, reduced SCFA levels, particularly butyrate, have been associated with increased disease risk, whereas butyrate itself has been described as an anti-proliferative and anti-tumorigenic metabolite. In addition, propionate has also been reported to inhibit CRC cell growth. By contrast, dysregulated secondary bile acids, especially deoxycholic acid and lithocholic acid, have been linked to increased CRC risk, whereas ursodeoxycholic acid has shown preventive effects in some animal and preclinical studies, although its role may be context-dependent (Hulin et al., 2025). Dietary Trp is metabolized by the gut microbiota into derivatives such as indole-3-propionic acid (IPA), indole-3-acetic acid (IAA), and indole-3-lactic acid (ILA). These metabolites function as ligands for the aryl hydrocarbon receptor (AhR), which is critical for the maintenance of intestinal immune homeostasis and barrier function (Cheng et al., 2022). In advanced CRC, Trp metabolism has been reported to shift toward the kynurenine pathway, accompanied by reduced indole-related metabolites, suggesting disruption of microbiota-associated homeostatic signaling and a transition toward a more immunosuppressive metabolic profile (Hulin et al., 2025).

The structural and functional integrity of the intestinal mucosal barrier is essential for defense against damage from gut microbiota dysbiosis and their metabolites. This barrier, composed of intestinal epithelial cells (IECs) and tight junctions (TJs) including zonula occludens-1 (ZO-1) and occludin, prevents pathogen entry and maintains homeostasis (Tang et al., 2022; Wei et al., 2023; He et al., 2024; Li et al., 2024b; Zhou et al., 2024). Secretory mucins from goblet cells, particularly Mucin 2 (Muc2), form a protective layer; reduction of goblet cells in UC patients decreases mucin synthesis, exacerbating inflammation (Gersemann et al., 2009; Okumura and Takeda, 2024). Muc2 knockout mice display colitis and adenomas progressing to invasive tumors, highlighting that sustained mucus barrier dysfunction contributes to dysplasia and colorectal tumorigenesis (Van der Sluis et al., 2006). Impairment of the intestinal barrier induces bacterial translocation. Specifically, loss of the mucus layer leads to adhesion of the microbiota to the epithelium, thereby accelerating the process of bacterial translocation (Grootjans et al., 2013; Kiely et al., 2018). Once the epithelial barrier is compromised, bacterial products can activate pattern-recognition receptor pathways, particularly TLR2/TLR4-and NF-κB-related signaling, thereby sustaining a pro-inflammatory microenvironment that favors malignant transformation (Sheflin et al., 2014). Indole metabolites of Trp activate AhR, enhancing interleukin-22 (IL-22) secretion, upregulating tight junction proteins and MUC2, and inducing Treg differentiation, thereby reinforcing barrier integrity and immune homeostasis (Jiang et al., 2025). Therefore, restoring gut microbiota homeostasis and intestinal barrier integrity may represent not only a pivotal therapeutic strategy for UC, but also a potential preventive approach against inflammation-associated colorectal tumorigenesis.

### 2.3 Oxidative stress and redox imbalance in the UC–CRC continuum

Oxidative stress, initially defined by Helmut Sies in 1985 (Sies, 2020), is characterized by an imbalance between oxidants and antioxidants. It has emerged as a critical factor in the pathogenesis of IBD and is closely associated with disease activity, progression, and clinical outcomes (Tratenšek et al., 2024). Although the precise pathogenesis of IBD is not fully elucidated, it potentially involves interplay between multiple factors, including the overproduction of reactive oxygen species (ROS), damage to biomolecules, mitochondrial dysfunction, immune cell recruitment, impairment of antioxidant defense systems, and activation of inflammatory pathways (Muro et al., 2024). Under physiological conditions, aerobic metabolism generates ROS primarily in the mitochondria. However, overproduction of ROS disrupts cellular homeostasis, leading to severe oxidative damage (Jakubczyk et al., 2020). Mitochondria play a pivotal role in cellular oxidative metabolism. Inflammation significantly induces intracellular ROS generation and causes mitochondrial structural alterations, leading to mitochondrial dysfunction and swelling, ultimately culminating in cell death. ROS are predominantly derived from impaired mitochondria and ultimately disrupt the intracellular homeostasis and exacerbate cellular injury (Kuznetsov et al., 2022; Wen et al., 2023; Li N. et al., 2024). Inflammation is frequently accompanied by oxidative stress, leading to severe disruption of the colonic mucosa, coupled with infiltration of inflammatory cytokines that trigger ROS generation (Wang Y. et al., 2023). Under conditions of chronic intestinal inflammation, activation of immune cells such as macrophages and neutrophils generates excessive ROS and reactive nitrogen species. These species induce both oxidative stress (OS) and nitrosative stress, which subsequently trigger a cascade of deleterious reactions such as lipid peroxidation, protein modification, DNA damage, and carcinogenic cellular transformation (Piechota-Polanczyk and Fichna, 2014; Tian et al., 2017; Guan and Lan, 2018; Bourgonje et al., 2020; Tratenšek et al., 2024). Ultimately, these processes lead to tissue injury, impaired mucosal healing, and persistence of inflammation, while also contributing to colorectal carcinogenesis.

Persistent overproduction of reactive oxygen and nitrogen species (ROS/RNS) may promote genomic instability and facilitate the accumulation of carcinogenic alterations during the transition from UC to CRC (Roessner et al., 2008; Piechota-Polanczyk and Fichna, 2014). Under the chronic inflammatory conditions described above, sustained ROS accumulation can induce oxidative cellular damage at early stages of carcinogenesis, particularly DNA damage, thereby promoting mutagenesis and malignant transformation of normal epithelial cells. This represents a critical initiating event in inflammation-associated tumorigenesis. In addition to causing direct mucosal injury, ROS also function as signaling molecules involved in cell-cycle regulation. ROS can induce p53 expression and lead to S-phase arrest, whereas epithelial Nrf2 deficiency results in oxidative stress and DNA damage accompanied by G2/M-phase arrest. These findings suggest that redox imbalance may disrupt epithelial homeostasis through cell-cycle dysregulation and thereby influence tumor development (Piechota-Polanczyk and Fichna, 2014). Moreover, experimental evidence has shown that limiting oxidative DNA damage can reduce microbe-induced CAC, further supporting a role for oxidative stress in the progression from chronic intestinal inflammation to malignancy (Irrazabal et al., 2020). Accumulated ROS may also act as chemical messengers to activate signaling pathways such as NF-κB and p38 MAPK, thereby influencing cell proliferation, differentiation, and apoptosis. In addition to oxidative DNA damage, carbonyl compounds generated by lipid peroxidation may further exacerbate mucosal injury and carcinogenic progression. Collectively, these findings indicate that oxidative stress is not merely a consequence of inflammation, but may also actively participate in inflammation-associated tumorigenesis (Wang et al., 2016). The Kelch-like ECH-associated protein 1 (Keap1)/nuclear factor erythroid 2-related factor 2 (Nrf2) pathway is a canonical oxidative stress response pathway and exhibits a clear context-dependent dual role in CRC. During the early stages of colorectal carcinogenesis, Nrf2 activation appears to be predominantly protective. Nrf2 can attenuate oxidative stress and inflammation and suppress ROS-induced intestinal injury, thereby helping to prevent CRC development. In parallel, Nrf2 upregulates Heme Oxygenase-1 (HO-1) expression, which further inhibits the nuclear translocation of NF-κB p65 and reduces the levels of pro-inflammatory mediators such as TNF-α, IL-1β, IL-6, and Cyclooxygenase-2 (COX-2). However, Nrf2 overexpression or aberrant activation has also been associated with tumor growth, malignant progression, chemoresistance, and poor prognosis in CRC (Bardelčíková et al., 2023).

Antioxidants play a pivotal role in maintaining systemic redox homeostasis by mitigating the impact of ROS. They protect cells from harmful and unstable molecules, thereby preventing oxidation of both endogenous and exogenous components. Intracellular endogenous antioxidants are categorized into enzymatic antioxidants such as superoxide dismutase (SOD), catalase (CAT), and peroxidases, and non-enzymatic antioxidants such as tocopherols, glutathione, and ascorbic acid (Irato and Santovito, 2021). In the category of natural antioxidants, the reduced form of glutathione (GSH) functions primarily to scavenge reactive oxygen intermediates and free radicals generated during metabolism. CAT catalyzes the decomposition of hydrogen peroxide and prevents *in vivo* OS. GSH participates in ROS scavenging to protect the organism from oxidative damage. Malondialdehyde (MDA) is a key indicator of lipid peroxidation. Colonic tissues from mice with UC and CAC exhibit reduced levels of CAT and GSH, accompanied by elevated levels of MDA (Li N. et al., 2024). This confirms that OS plays a pivotal role in the pathogenesis of UC and CRC. Furthermore, the Keap1–Nrf2–antioxidant response element (ARE) pathway acts as the most critical cellular defense mechanisms against OS, protecting various organs and cells from damage induced by exogenous substances. These findings suggest that targeting the Nrf2/Keap1 signaling pathway may offer a potential therapeutic strategy to alleviate intestinal inflammation in patients with UC and CRC (Linghu et al., 2024; Ma et al., 2025).

### 2.4 Cell death regulation in intestinal disease progression

In recent years, increased understanding of the pathophysiology of UC has resulted in identification of new mechanistic targets for therapeutic intervention. The physiological death of IECs is required for the maintenance of intestinal function, sustained renewal capacity, and tissue homeostasis. Conversely, aberrant IECs death causes erosion and injury of the intestinal epithelial, leading to the onset of UC (Long et al., 2024). Ferroptosis is a form of programmed, iron-dependent cell death, with iron metabolism and lipid peroxidation signaling serving as its key mediators (Chen et al., 2020). Ferroptosis is intricately associated with IECs death in UC (Wang et al., 2020; Xu M. et al., 2020; Chen Y. et al., 2022). During UC, depletion of reduced GSH, inactivation of glutathione peroxidase 4 (GPX4), and lower levels of antioxidant factors such as Nrf2 compromise the defensive capacity of the antioxidant system and exacerbate oxidative stress-induced injury (Chen et al., 2021b; Dong et al., 2021; Chen L. et al., 2022). Concurrently, ferroptosis involves uncontrolled lipid peroxidation, leading to the accumulation of ROS and MDA, and leads to damage of the mucosal barrier. In the inflamed regions associated with UC, high levels of hydrogen peroxide (H_2_O_2_) and iron ions released from hemoglobin degradation further exacerbate this process. Iron overload and uncontrolled ROS, coupled with the impairment of the antioxidant system, directly induce IECs death, increase intestinal permeability, and compromise the physical structure of the intestinal mucosal barrier. Subsequently, the invasion of bacteria and toxins triggers chronic inflammation. Furthermore, suppression of the antioxidant system disrupts regenerative capacity and homeostasis of the IECs, thereby establishing a vicious cycle (Millar et al., 2000; Chen et al., 2020; Wang et al., 2020; Xu M. et al., 2020; Chen L. et al., 2022; Huang et al., 2022; Sun et al., 2023; Yokote et al., 2023; Long et al., 2024). Ferroptosis-driven IECs death not only exacerbates acute inflammation but also leads to intestinal fibrosis and CRC through persistent barrier dysfunction (Long et al., 2024).

Ferroptosis is increasingly recognized as a critical mechanistic nexus driving the progression from UC to CRC, particularly CAC. In the setting of persistent intestinal inflammation, disruption of local iron homeostasis, excessive accumulation of ROS, and enhanced lipid peroxidation collectively induce ferroptosis, marked by impairment of the GPX4-dependent antioxidant defense system, thereby leading to intestinal epithelial cell loss and disruption of mucosal barrier integrity. Once the barrier is compromised, luminal bacteria and their associated products penetrate the mucosa more readily, exacerbating microbial dysbiosis, immune cell infiltration, and inflammatory responses, establishing a vicious cycle of epithelial injury, barrier disruption, and amplification of inflammation. Increased macrophage infiltration and M1 polarization, sustained release of pro-inflammatory cytokines such as IL-1β, IL-6, and TNF-α, and activation of protein kinase B (AKT)/nuclear factor kappa-B kinase (IKK)/P65, extracellular Signal-Regulated Kinase (ERK)/IKK/P65, and NF-κB-related signaling pathways contribute to chronic inflammation. Meanwhile, the ROS burst and accumulation of lipid peroxidation products, including MDA, aggravate tissue injury and may induce oxidative DNA damage, epigenetic abnormalities, and pro-tumorigenic signaling reprogramming, creating conditions conducive to aberrant epithelial proliferation and tumor microenvironment formation. Studies in dextran sulfate sodium (DSS)-induced colitis models and azoxymethane/dextran sulfate sodium (AOM/DSS)-induced CAC models consistently show that inhibition of ferroptosis alleviates colitis severity, reduces iron accumulation and lipid peroxidation, decreases pro-inflammatory cytokine release, and suppresses tumor burden and growth (Tang et al., 2021; Liang J. et al., 2024; Long et al., 2024). Collectively, these findings indicate that ferroptosis is not merely an epiphenomenon in UC and CRC, but rather a key driving force that continuously promotes the transition from UC to CRC, especially CAC, by linking oxidative stress, mucosal barrier disruption, dysregulated immune inflammation, and remodeling of a pro-carcinogenic microenvironment.

As an evolutionarily conserved intracellular degradation and recycling system, autophagy plays a pivotal role in maintaining intestinal homeostasis. Recent studies have indicated that autophagy dysfunction is intricately linked to the pathogenesis of UC. It participates in disease progression through multiple dimensions, including modulation of gut microbiota composition and regulation of immune responses (Larabi et al., 2020). A bidirectional regulatory relationship exists between autophagy and the gut microbiota. Defects in autophagy induce gut dysbiosis, characterized by the expansion of pro-inflammatory bacteria and a reduction in beneficial bacteria, thereby disrupting microecological balance (Tsuboi et al., 2015; Yang L. et al., 2018; Lavoie et al., 2019; Larabi et al., 2020). Furthermore, microbial metabolites such as SCFAs modulate host autophagy activity (Donohoe et al., 2011). Conversely, pathogenic bacteria such as adherent-invasive *Escherichia coli* (AIEC) inhibit the expression of autophagy-related genes by upregulating molecules like miR-30c and miR-130a, thereby enabling them to evade intracellular clearance and exacerbate inflammatory responses (Lu et al., 2014; Bretin et al., 2018). Furthermore, autophagy influences multiple facets of both innate and adaptive immunity, including facilitating intracellular pathogen clearance and regulating antigen presentation as well as T cell differentiation and homeostasis. Notably, risk variants such as ATG16L1 T300A induce immune cell dysfunction, promote Th1/Th17 responses, and disrupt immune homeostasis, thereby driving the progression of UC (Yang L. et al., 2018; Larabi et al., 2020). Studies have shown that autophagy plays a marked stage-dependent dual role in CAC initiation and progression. During inflammation-driven malignant transformation, autophagy suppresses CAC initiation by maintaining intestinal epithelial barrier integrity, preserving Paneth cell and intestinal stem cell homeostasis, attenuating ROS accumulation and endoplasmic reticulum (ER) stress, limiting activation of the NOD-like receptor family pyrin domain containing 3 (NLRP3) inflammasome and necroptotic and pyroptotic signaling, and promoting pathogen clearance and intestinal microbiota stabilization. These effects reduce chronic inflammation, DNA damage, and aberrant regeneration. Conversely, in established CRC, particularly CAC, cytoprotective autophagy supports tumor cell metabolism and tolerance to hypoxia, radiotherapy, and chemotherapy, thereby sustaining tumor growth and therapeutic resistance (Wu et al., 2018; Jin et al., 2024).

Overall, dysregulated cell death is a critical driver of intestinal disease progression in the UC–CRC continuum. In particular, ferroptosis promotes inflammation-associated carcinogenesis by linking oxidative stress to barrier disruption and pro-tumorigenic remodeling, whereas autophagy exerts a stage-dependent dual role, suppressing early malignant transformation but supporting survival and therapeutic resistance in established CRC/CAC.

## 3 Botanical drugs and representative metabolites of GQD under the same mechanistic framework

**FIGURE 2:**
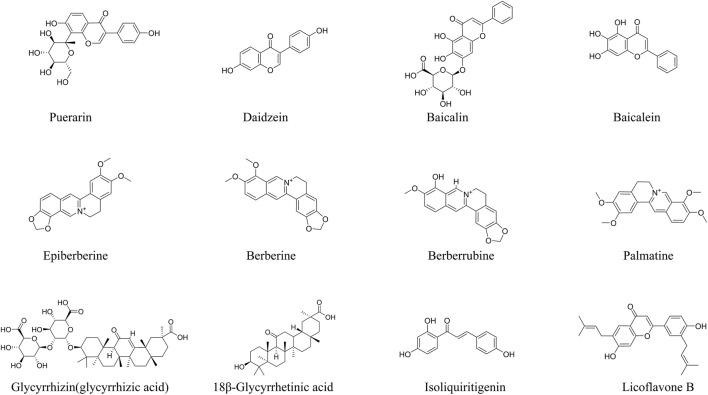
Molecular structures of the representative bioactive metabolites derived from GQD.

GQD is composed of four medicinal botanical drugs: *Puerariae Lobatae Radix* (Gegen), *Scutellariae Radix* (Huangqin), *Coptidis Rhizoma* (Huanglian), and *Glycyrrhizae Radix et Rhizoma* (Gancao). A growing body of evidence suggests that bioactive metabolites derived from these botanical drugs exhibit notable anti-inflammatory and antioxidant activities, as well as potential protective effects on the intestinal mucosal barrier (Supplementary Table 1). In addition, some of these metabolites have been reported to show antitumor activity (Table 2). The molecular structures of the relevant bioactive metabolites are shown in Figure 2.

**TABLE 2:** Summary of antitumor studies on representative bioactive metabolites of GQD.

Botanical drug & validated taxon (Family)	Metabolite	Experimental model (*In vivo*/*In vitro*)	Dose range/Concentration & duration	Controls used (Positive/Negative)	Key pharmacological mechanisms	Critical assessment & limitations	Ref.
Pueraria Montana var. lobata (willd.) maesen & S.M.Almeida ex sanjappa & Predeep (Fabaceae)	Puerarin	*In vivo*: AOM/DSS-induced CAC in male BALB/c mice. *In vitro*: pH-dependent release/swelling and GI retention assays	Puerarin (>99%), pH-responsive alginate microspheres. *In vivo*:4, 6, 8 mg/kg, p.o., qod (day 42–63).Free puerarin: 8 mg/kg AOM 10 mg/kg + 2.5% DSS; sustained colonic release/retention assessed	Positive control: Salicylazosulfapyridine (SASP, 450 mg/kg). Formulation control: Free puerarin (8 mg/kg).Disease model: AOM/DSS + vehicle. Negative control: 0.9% saline	Colon-targeted release and retention. ↓ TNF-α, IL-17A, TGF-β1, COX-2 (mRNA). EMT-related: ↑ E-cadherin; ↓ N-cadherin, Snai1, Zeb1, Twist1, MMP-9	Relevant CAC model with positive/formulation controls; limitations: mRNA-level evidence only, no *in vitro* pharmacology, unclear minimum effective dose, male-only design, and unresolved formulation–drug contribution	Deng et al. (2019)
	Daidzein	*In vivo*: None. *In vitro*: TNF-α-stimulated ER-negative MCF10DCIS.com human breast cancer cells. Supplementary mechanistic model: Ptch1(−/−) MEFs	Daidzein(no purity) *In vitro*: Daidzein at 30 μM (non-toxic concentration); TNF-α stimulation at 5 ng/mL; co-incubation for 24 h.equol and 6,7,4′-trihydroxyisoflavone also examined	Positive controls: Specific Hedgehog (Hh) signaling inhibitors; Gli1 overexpression/siRNA. Disease model: TNF-α stimulated cells. Negative control: Untreated vehicle cells	Daidzein was associated with reduced Hh/Gli1 activation, TNF-α-induced migration/invasion, and MMP-9 expression/activity, with limited effect on MMP-2	Functional and Gli1-perturbation assays support pathway involvement; Limitations: *In vitro* only; no *in vivo* validation; purity not reported; mainly one cancer cell line	Bao et al. (2014)
Scutellaria baicalensis Georgi (Lamiaceae)	Baicalin	*In vivo*: Orthotopic HCT116-GFP CRC model; *In vitro*: Human colorectal cancer cell lines (FHC, RKO, HCT116); HCT116-derived 3D spheres	Baicalin (98% purity, National Institute for the control of Pharmaceutical and biological product, China). *In vivo*: 100, 200 mg/kg, i.g.,4 weeks *In vitro*: 50, 100 μg/mL for 48 h	Positive control: 5-FU. Disease model: Orthotopic HCT116-GFP colorectal tumors; EMT models Negative control: DMSO/vehicle controls	Induces cell cycle arrest at G1 phase and inhibits TGFβ/Smad pathway; ↓expression of EMT and stemness proteins; ↓cell growth, migration, and proliferation; induces apoptosis	Combined *in vitro* and orthotopic *in vivo* validation with 5-FU control; limitations: Incompletely specified *in vitro* dosing and unresolved upstream regulation of TGF-β/Smad signaling	Yang et al. (2020)
​	​	*In vivo*:Male CT26 xenograft model; *In vitro*: SW480/HCT-116/CT26 cells; *In silico*: Bioinformatic analysis (StarBase/ENCORI, TargetScan)	Baicalin (MedChemExpress, Shanghai, China; purity 98.01%). *In vivo*: Baicalin 50 mg/kg, i.p., daily for 3 weeks *In vitro*: 50/100/150/200 μg/mL for 24 h or 7 days	Positive control: None. Disease model: CT26 xenograft-bearing mice; SW480/HCT-116 cells. Negative control: DMSO-treated cells; sham xenograft mice. Other controls: miR-139-3p/CDK16 gain- or loss-of-function	↑miR-139-3p and ↓CDK16, thereby inhibiting proliferation and inducing S-phase arrest; miR-139-3p directly targets CDK16; xenograft inhibition is enhanced by CDK16 knockdown	*In vitro* and xenograft data with miR-139-3p/CDK16 perturbation support the axis; limitations: No positive control, subcutaneous model, and incomplete *in vivo* mechanistic validation	Cai et al. (2023)
		*In vivo*: Subcutaneous xenograft model of human colon cancer (HCT116 cells) in athymic BALB/c nude mice. *In vitro*: HCT116/SW480 cells, as well as A549 and Panc-1 lines	Baicalin (purity >93%, Jiangsu Institute for food and drug Control). *In vivo*: 80 mg/kg, i.p. (intraperitoneal injection), daily for 14 days (starting on day 5 after tumor inoculation.). *In vitro*: “Various concentrations” for 24 h up to 2 weeks	Positive control: None. Mechanistic control: Scrambled non-targeting siRNA vs. DEPP-specific siRNA. Negative control: Untreated cells, and xenograft controls	Induces cellular senescence without obvious apoptosis. ↓ ROS; ↑ SOD activity.↑ DEPP expression.Activates Ras/Raf/MEK/ERK and p16INK4A/Rb signaling. DEPP knockdown attenuates growth inhibition, S-phase arrest, and senescence	*In vitro*/*in vivo* models and DEPP perturbation support senescence-related effects; limitations: No anticancer control, non-oral dosing, and absence of orthotopic or inflammation-associated CRC models	Wang et al. (2018)
		*In vivo*: None. *In vitro*: Human colon cancer cell lines DLD1 and HCT-116	Baicalin (weikeqi, sichuan, China; purity not specified). *In vitro*: Baicalin 0–40 μM for 24 h or 6 days. Luciferase assay: miR-217 mimic/inhibitor, 50 nM, for 48 h	Positive control: None. Disease model: Baicalin-treated DLD1/HCT-116 colon cancer cells. Negative control: Untreated/vehicle-treated cells; siRNA/mimic/inhibitor controls, and luciferase vector controls	Induces apoptosis and inhibits proliferation/colony formation. ↑ DKK1; ↓ β-catenin, c-Myc.↓ miR-217. Inhibits wnt/β-catenin signaling via the miR-217/DKK1 axis	Dose-response, colony, knockdown and luciferase assays support the proposed axis; limitations: *in vitro*-only evidence, unreported purity, no positive control, and two-cell-line validation	Jia et al. (2019)
​	​	*In vivo*: Subcutaneous HT-29 xenograft model in 6-week-old male nude mice. *In vitro*: HT-29 cells with SW-480/CACO2 validation. *In silico*: miRNA microarray with GO/KEGG analysis	Baicalin (sigma; no purtiy). *In vivo*: 50, 100 mg/kg, i.p., daily for 3 weeks (started 1 week post-inoculation). *In vitro*: 0–600 µM for 24 h or 150 µM for up to 48 h (viability assay); 50–200 µM for 48 h (apoptosis assay)	Positive controls: Pan-caspase inhibitor (Z-VAD-FMK); c-myc siRNA/overexpression, miRNA mimics/inhibitors. Disease model: Subcutaneous HT-29 xenograft + Vehicle. Negative control: Vehicle	Induces apoptosis and inhibits tumour growth. ↓ c-myc and several oncomiRs. Upregulates apoptosis-related targets (PDCD4, HIC1, PTEN, E2F2, E-cadherin) and ↓ BCL-2	Integrates xenograft, miRNA profiling and c-myc perturbation; limitations: No standard positive control, subcutaneous/non-oral model, and unreported purity	Tao et al. (2018)
	Baicalein	*In vivo*: Subcutaneous CRC xenograft model in female BALB/c nude mice. *In vitro*: Human CRC cell lines HCT116 and DLD1; normal colonic epithelial cell line NCM460 for toxicity comparison	Baicalein (MedChemExpress, Shanghai, China; no purity). *In vivo*: 10/20 mg/kg, i.g., every 2 days for 2 weeks *In vitro*: 1–64 μM (for viability); 7.5–30 μM (for mechanism assays)	Positive control: None. Negative control: Vehicle/normal solvent; untreated cells. Other controls: Lip-1 ferroptosis inhibitor; chloroquine, necrostatin-1, Z-VAD-FMK as non-ferroptosis cell-death controls; STAT3 overexpression control	Promote ferroptosis through JAK2 targeting and ↓JAK2/STAT3/GPX4. ↑ ROS, MDA, Fe2+; ↓ GSH, MMP, GPX4, NRF2, HO-1, FTH1; ↑ TFR1. Effects were mitigated by Lip-1 or STAT3 overexpression	*In vitro* + xenograft validation with ferroptosis rescue and JAK2 target-binding evidence. Limitations: No positive control; purity not reported	Lai et al. (2024)
Coptis chinensis Franch., Coptis deltoidea C.Y.Cheng & P.K.Hsiao, or Coptis teeta wall. (Ranunculaceae)	Berberine	*In vivo*: CAC model induced by AOM/DSS in male C57BL/6J mice; fecal microbiota transplantation (FMT) validation model. *In vitro*: None	Berberine (no source/purity). *In vivo*: 50, 100 mg/kg b.wt./day, p.o., daily for 84 days *In vivo* (FMT): FMT material administered orally at 10 mL/kg, twice a week for 10 weeks	Positive control: Aspirin (50 mg/kg/day).Negative control: Normal mice; solvent-treated AOM/DSS mice. Other controls: FMT donor-recipient comparison	Berberine was associated with microbiota remodeling, increased SCFAs, reduced fecal LPS, improved occludin/ZO-1, and lower TLR4/p-NF-κB p65/IL-6/p-STAT3 signaling; FMT partially reproduced effects	AOM/DSS, 16S, SCFA profiling, FMT and aspirin comparator strengthen interpretation; limitations: *in vivo*-only design, unreported source/purity, and lack of direct target validation	Yan et al. (2022)
		*In vivo*:AOM/DSS colitis and 84-day CAC models in C57BL/6J male mice *In vitro*: None	Berberine (BBR) (no purity and manufacturer). *In vivo*: 100 mg/kg, p.o. (oral administration/gavage), daily for 18 days (colitis model) or 84 days (CAC model)	Positive control: None.Disease model: AOM/DSS + PBS vehicle (CAC group). Negative control: PBS daily (CON group)	↓ TNF-α, IL-1β; ↑ IL-10; improves barrier function. ↓ tumor burden, ↓ Ki-67, ↑ apoptosis. ↑ beneficial taxa, ↑ SCFAs; associated with ↓ wnt-related signaling	Disease-relevant colitis/CAC models with histology, barrier, microbiome, transcriptomic, metabolomic and SCFA analyses; limitations: *in vivo*-only design, no positive control, unreported purity, and mainly associative mechanisms	Wang et al. (2024c)
​	​	*In vivo*: AOM/DSS-induced CAC in male C57BL/6 mice; contrast CRC (without chronic colitis). *In vitro*: LPS-induced RAW264.7 macrophages; LPS/IFNγ-stimulated bone marrow-derived macrophages (BMDMs)	Berberine (solarbio, China; no purity). *In vivo*:40 mg/kg, oral gavage, every other day. *In vitro*: Berberine 5, 10, 20, 40 μM; LPS stimulation in RAW264.7; LPS/IFNγ stimulation in BMDMs.miR-155-5p/SOCS1 perturbation	Positive control: None.Disease model: AOM/DSS-induced CAC; AOM + single DSS CRC model; LPS- or LPS/IFNγ-stimulated macrophages. Negative control: Vehicle-treated mice; untreated/control cells	Inhibits CAC in an inflammation-dependent manner. ↓ macrophage infiltration and M1 polarization; ↓ IL-1β, IL-6, TNF-α, MCP-1, NO. ↓ miR-155–5p; ↑ SOCS1; ↓ p-STAT1	CAC/CRC comparison and macrophage validation with miR-155-5p/SOCS1 perturbation support the pathway; limitations: No positive control, unreported purity, and incomplete *in vivo* pathway validation	Ling et al. (2023)
		*In vivo*: DSS-treated ApcMin/+ CAC model. *In vitro*: RAW 264.7 macrophages; IMCE cells; HCT116 cells	Berberine(Sigma Aldrich, St. Louis, MO, USA; no purity). *In vivo*: Berberine 1 mg/mL in drinking water for 4 weeks; additional 50 mg/kg i.g. schedule with IL-6 rescue. *In vitro*: 25 μM with LPS-conditioned medium	Positive control: None. Negative control: Normal drinking water; PBS vehicle; untreated/control cells. Other controls: DSS + IL-6 and DSS + Ber + IL-6 groups	Berberine was associated with reduced macrophage/colon IL-6 and TNF-α, decreased EGFR/ERK signaling and epithelial proliferation; IL-6 partly attenuated the effect	ApcMin/+ CACRC model with macrophage–epithelial assays and IL-6 rescue supports the proposed axis; limitations: No positive control, unreported purity, and preclinical pathway-focused evidence	Li et al. (2017)
	Palmatine	*In vivo*: Subcutaneous tumor xenografts using HCT116 (wild-type and MSI2-knockout) cells in immunodeficient male BALB/c nude mice. *In vitro*: HCT116, A549 and HEK293FT cells with CRISPR MSI2 knockout	Palmatine chloride (MedChemExpress, HY-N0110; no purity). *In vivo*: 50 mg/kg/day, i.p., for 13 days *In vitro*: Palmatine 0.25–64 μM for 72 h (CCK8); with CETSA/RIP-qPCR and comparator compounds	Positive control: None.Negative control: PBS or vehicle-treated mice; DMSO/PBS-treated cells; GFP control in MST; IgG control in RIP-qPCR. Other controls: MSI2-knockout lines, Ro 08-2750 and (−)gossypol comparators	Directly binds MSI2,→inhibits MSI2-dependent CRC cell growth. ↓MSI2 RNA-binding to targets (KIF18A, ZFHX4, PEG10, EIF4EBP1). MSI2 depletion causes G0/G1 arrest with ↓ cyclin E1, without increased apoptosis	MSI2 knockout/rescue, binding assays, RIP-qPCR and xenografts support MSI2 involvement; limitations: No positive control, subcutaneous/i.p. model, possible MSI1/RBP binding, and early weight loss at 50 mg/kg/day	Zhang et al. (2022)
​	​	*In vivo*: AOM/DSS-induced CRC in male C57 mice; HCT116 xenograft models in male mice.Human CRC tissue microarray. *In vitro*: HCT116/SW480 and 293T cells	Palmatine (Zhengzhou Alfa chemical co., LTD, Zhengzhou, China; purity >98%). *In vivo*: PAL 33.75, 67.5, 135 mg/kg, gavage, once daily. *In vitro*: PAL 352–354 μM for 24 h with CHX/CQ/MG132, CETSA and DARTS assays	Positive control: None. Negative control: Blank/control mice; blank plasmid controls (pCDH, pcdna3.1, pSGU6); untreated cells. Other controls: AURKA overexpression, AURKA-NLS overexpression, MYH9 overexpression/knockdown; CHX, CQ, MG132	Palmatine was associated with reduced AURKA, lysosomal autophagy-dependent AURKA degradation, decreased nuclear AURKA via AURKA–MYH9 modulation, and G2/M arrest with lower CCNB1/CDK1/p-H3(S10)	Patient tissue, AOM/DSS, xenograft and cell data support AURKA/MYH9 involvement. Limitations: No positive control, no direct biophysical binding assay, and focus on nuclear AURKA.	Tang et al. (2024b)
	Berberrubine	*In vivo*: SW620 xenografts, AOM/DSS spontaneous CRC model(female mice *In vitro*: Human CRC cell lines SW620, LS174T, SW480, RKO, and COLO205. Colony formation and 3D soft agar assays. *In silico*: Docking and virtual screening	Berberrubine (TargetMol, MA, USA; no purity). *In vivo*: 25 or 50 mg/kg/day, p.o.; xenograft for 18 days; AOM/DSS to 100. *In vitro*: Mainly 10–30 μM with guanosine rescue	Positive control: Mycophenolic acid in enzyme screening assay. Negative control: DMSO vehicle; CMC-Na vehicle *in vivo*. Other controls: Guanosine rescue (200 μM) in colony/3D assays	Directly and selectively inhibits IMPDH2, blocks *de novo* guanine synthesis, and suppresses CRC growth; effects are guanosine-rescuable, with reduced tumor burden and Ki67 *in vivo*	Enzymatic selectivity, target-binding, guanosine rescue, xenograft/AOM-DSS and clinical tissue/database support are included; limitations: Unreported purity, no standard anticancer comparator, and unexcluded off-targets	He et al. (2023)
Glycyrrhiza uralensis Fisch. ex DC., Glycyrrhiza inflata Batalin, or Glycyrrhiza glabra L. (Fabaceae)	glycyrrhizic acid	*In vivo*: AOM/DSS-induced CAC mice; PAD4^−/−^ CAC mice; human UC/CAC-related biopsy analysis. *In vitro*: PMA-induced NETosis in dHL-60 cells; NET supernatant-treated Caco-2 cells; CD8^+^ T cell–CT26 coculture	Glycyrrhizic acid (GA; Yuanye, China; no purity). *In vivo*: 10, 20 or 30 mg/kg/day, p.o.; AOM/DSS-induced CAC model for 9 weeks *In vitro*: 20, 40, 60 μM; GSK484 20 μM; PMA-induced NET formation for 4–6 h	Positive controls: GSK484 as PAD4 inhibitor; aspirin in CAC model; PAD4^−/−^ mice/neutrophil-derived NET supernatant.Negative/model controls: Normal, AOM/DSS model, PMA-induced NET model, untreated/vehicle conditions	GA was associated with reduced PAD4 activity and NET formation; ↓citH3, MPO-DNA, dsDNA, ROS, MPO and MMP-9. ↓tumor burden and Ki67, ↓neutrophil-associated inflammation, ↑ZO-1/occludin, and partially ↑CD8+ T cell–mediated tumor cytotoxicity	CAC model with PAD4 inhibitor, aspirin, PAD4^−/−^ and coculture validation. Limitations: Preclinical evidence, small biopsy cohort, PAD4–NET-focused mechanism, limited PK data, and no chemo/immunotherapy comparator	Chen et al. (2025c)
​	Isoliquiritigenin	*In vivo*: None. *In vitro*: Human CRC cell lines HT29 and HCT116	Isoliquiritigenin (INDOFINE chemical Company, Hillsborough, NJ, USA; no purity). *In vitro*: 10, 30, 50 μmol/L for 24 h	Positive control: Ionomycin. Negative control: DMSO vehicle control; untreated/control cells; OPSS calcium-free condition. Other controls: Capsazepine co-treatment as TRPV1 inhibition control	↑ cytosolic Ca2+ via extracellular influx and intracellular release. ↑ TRPV1 expression. Induces apoptosis via TRPV1. ↓ Bcl-2; ↑ Bax, Caspase-3, Caspase-9 mRNA.	Two-cell-line *in vitro* data with CapZ, calcium imaging, Western blot, flow cytometry and qPCR support TRPV1 involvement.Limitations: No *in vivo* validation, unreported purity, and no positive anticancer control	Wang et al. (2025b)

### 3.1 Puerariae lobatae radix (Gegen)

*Puerariae Lobatae Radix* (Gegen), the dried root of *Pueraria montana* var. *lobata* (Willd.) Maesen & S.M.Almeida ex Sanjappa & Predeep, possesses anti-inflammatory, antioxidant, vasodilatory, lipid-lowering, and hypoglycemic properties, and mitigates alcohol dependence (Gao et al., 2024). Modern pharmacological studies have identified isoflavones and isoflavone glycosides, such as puerarin, daidzein, β-sitosterol, stigmasterol, and arachidic acid as primary active metabolites (Lu et al., 2021; Gao et al., 2024).

Pharmacokinetic studies have demonstrated that puerarin and daidzein are representative metabolites of Puerariae Lobatae Radix, with puerarin being the most highly exposed metabolite in the colon (Lu J. Z. et al., 2022). In clean-grade mice, the colonic peak concentration of puerarin after oral GQD administration reached 61,585.3 ng/g. However, because this value represents the total concentration in tissue homogenates, it is still not possible to directly establish a correspondence between colonic puerarin exposure, free active concentration, and specific *in vitro* effective concentrations. As a major isoflavone, puerarin exhibits significant antitumor, anti-inflammatory, and antioxidant properties (Kapoor, 2013; Singh et al., 2013; Jeon et al., 2020; Hu Z. et al., 2024). Jeon et al. confirmed that puerarin protects against DSS-induced UC in mouse models by suppressing inflammation and OS, and regulating intestinal epithelial barrier function (Jeon et al., 2020). In UC, activated macrophages are significantly increased compared with controls and secrete pro-inflammatory cytokines that drive intestinal inflammation. Intestinal macrophages are essential for maintaining intestinal homeostasis and regulating inflammation resolution. Previous studies have shown that M1 macrophages cooperate with neutrophils to trigger Th1/Th17 immune responses, releasing IFN-γ, IL-17, and matrix metalloproteinases (MMPs), thereby forming a vicious pro-inflammatory cycle that causes tissue damage and barrier disruption. Puerarin directly inhibits M1 polarization and induces a shift toward the M2 phenotype, breaking this cycle. Furthermore, puerarin promotes tissue repair by upregulating major histocompatibility complex (MHC) molecules, as validated *in vitro* (Na et al., 2019; Wang K. et al., 2023; Tao et al., 2024). Animal studies have demonstrated that puerarin alleviates inflammation and pathological damage in UC mice by modulating the gut microbiota and repairing the colonic mucus barrier. In one study, UC mice exhibited dysbiosis of the intestinal flora characterized by a decreased F/B ratio, but puerarin intervention effectively restored this ratio and suppressed major pro-inflammatory taxa, thereby promoting the resolution of inflammation (Zou et al., 2023). Wu et al. further confirmed that puerarin promotes goblet cell differentiation and mucin secretion, thereby increasing mucus layer thickness and reducing colonic permeability to block bacterial erosion of the epithelium. Moreover, puerarin regulates composition of mucin-utilizing bacteria and elevates levels of SCFAs, thereby providing energy for epithelial repair and ultimately achieving functional remodeling of the colonic mucus layer (Wu et al., 2020). Chronic inflammation associated with UC is a pivotal driving factor in the “inflammation-to-cancer transition.” Activated immune cells produce pro-inflammatory cytokines such as TNF-α, IL-6, IL-17, and IL-23, which not only exacerbate the inflammatory response but also promote malignant transformation (Terzić et al., 2010). Consequently, these cytokines and related genes (including transforming growth factor-β1 (TGF-β1), COX-2, and inducible Nitric Oxide Synthase (iNOS)) frequently exhibit high expression in CAC (Axelrad et al., 2016). Puerarin-loaded alginate microspheres effectively intervene in this process. Mechanistically, they alleviate inflammatory injury by downregulating the mRNA expression of TNF-α and IL-17A. Concurrently, they exert antitumor and anti-metastatic effects by inhibiting epithelial-mesenchymal transition (EMT) in AOM/DSS-induced CAC mice (Deng et al., 2019).

Among the included puerarin-related studies, the dose range and experimental designs were heterogeneous. Jeon et al. provided preliminary dose-comparison evidence using 10 and 50 mg/kg/day oral puerarin in DSS-induced colitis, but a true minimum effective dose remains undefined because lower doses were not tested. Other oral colitis studies used 200 mg/kg/day as single-dose regimens, supporting pharmacological activity but not dose–response assessment, whereas intraperitoneal studies using 50–160 mg/kg/day provide mechanistic information with limited relevance for oral translational interpretation. Human equivalent dose conversion based on body-surface-area scaling provides only a rough translational reference and cannot by itself distinguish pharmacological from suprapharmacological effects (Blanchard and Smoliga, 2015). Clinically relevant effective exposure still requires validation through human pharmacokinetics, colonic tissue exposure assessment, and Pharmacokinetics–Pharmacodynamics (PK–PD) studies.

Daidzein is a natural isoflvone phytoestrogen belonging to the non-steroidal estrogen group, possessing antioxidant, anti-inflammatory, antitumor, and neuroprotective properties (Goleij et al., 2024). It significantly reduces the levels of core pro-inflammatory cytokines and chemokines, including IL-6, TNF-α, and monocyte chemoattractant protein-1 (MCP-1) (Sakamoto et al., 2014; Meng et al., 2017; Yu et al., 2020). Concurrently, daidzein promotes expression of anti-inflammatory mediators such as adiponectin and TGF-β, thereby restoring inflammatory homeostasis (Sakamoto et al., 2014; Yu G. et al., 2021). Numerous signaling pathways are involved in the anti-inflammatory effects of daidzein, among which mitogen-activated protein kinase (MAPK) and NF-κB pathways have been extensively investigated. Daidzein alleviates DSS-induced colitis in mice by modulating NF-κB signaling, leading to reduced levels of Myeloperoxidase (MPO), phosphorylated p65 Nuclear Factor kappa-B (p65-NF-kappaB), phosphorylated Inhibitor of NF-κB alpha(p-IκB-α), and phosphorylated IκB Kinase (p-IKK), as well as various inflammatory cytokines including TNF-α, IL-1 β, and IL-6 in colonic tissues (Shen et al., 2019b). Animal experiments have confirmed that daidzein regulates NF-κB, p38 MAPK, and TGF-β1 pathways to counteract angiotensin II-induced inflammation in mice (Liu et al., 2016). Regarding its antioxidant properties, daidzein promotes migration of free radicals by enhancing membrane fluidity (Liang et al., 2008). It also activates endogenous antioxidant enzymes such as CAT, SOD, and glutathione peroxidase (GPx), thereby inhibiting lipid peroxidation (Röhrdanz et al., 2002; Kampkötter et al., 2008; Dwiecki et al., 2009). Furthermore, *in vivo* metabolites such as 3′-OH-daidzein and 6-OH-daidzein are more potent antioxidants (Liang et al., 2008; Choi and Kim, 2014). Similarly, daidzein demonstrates distinct antitumor potential. TNF-α promotes tumor cell invasiveness and the expression of metalloproteinase-9 (MMP-9) mRNA. However, treatment with daidzein blocks activation and expression of glioma-associated oncogene homolog 1 (Gli1), a key effector in the Hedgehog (Hh) signaling pathway. This blockage subsequently downregulates the levels of TNF-α, which in turn suppresses downstream MMP-9 expression, ultimately reducing the invasive capability of cancer cells (Bao et al., 2014).

### 3.2 Scutellariae radix (Huangqin)

Scutellariae Radix (Huangqin) was first recorded in *Shennong Ben Cao Jing* and is derived from the root of *Scutellaria baicalensis* Georgi, a member of the Lamiaceae family (Shang et al., 2010). To date, more than 40 metabolites have been isolated and identified from Huangqin, including flavonoids, terpenoids, volatile oils, and polysaccharides (Zhao et al., 2019). Modern clinical studies have demonstrated its therapeutic efficacy for various diseases such as UC (Zhang J. et al., 2025), hepatitis (Zhao et al., 2016), respiratory tract infections (Luo Y. et al., 2024), diarrhea (Tang Z. W. et al., 2024), digestive system tumors (Zhao et al., 2024), pregnancy-related diseases (Fang et al., 2023), and cardiovascular diseases (Tan et al., 2022). Baicalin and baicalein are the primary chemical metabolites of Scutellariae Radix.

In recent years, immunomodulation has emerged as a viable option for UC treatment (Le Berre et al., 2023). Treg cells are essential for immune balance and tolerance. Through expression of the transcription factor forkhead box P3 (Foxp3), they maintain immune tolerance and suppress food allergies and autoimmune responses. Thus, inducing CD4^+^ Foxp3+ Treg differentiation has become a key strategy to regulate abnormal immune responses and treat related diseases (Dinda et al., 2017). Baicalein promotes differentiation of naive CD4^+^ T cells into CD4^+^Foxp3^+^Treg cells with immunosuppressive functions by activating the AhR signaling pathway. Simultaneously, it inhibits cytokines associated with Th1, Th2, and Th17 cells to rectify immune imbalance and alleviate allergic inflammatory responses. These effects collectively exert a synergistic role in dual regulation, which involves modulating the immune response and adjusting TJs to strengthen the intestinal physical barrier (Bae et al., 2016). An animal study showed that baicalin upregulates interferon regulatory factor 4 (IRF4) and downregulates interferon regulatory factor 5 (IRF5) to modulate macrophage polarization. This promotes the shift from pro-inflammatory M1 to anti-inflammatory M2 macrophages, effectively alleviating DSS-induced colitis in mice and offering a potential therapeutic approach for UC. In LPS-stimulated macrophages, baicalin decreased TNF-α, Interleukin-23 (IL-23), and IRF5 while increasing IL-10, Arginase-1 (Arg-1), and IRF4 (Zhu et al., 2016).

Oxidative stress represents a critical risk factor in the progression of chronic inflammatory diseases and serves as a primary driver of inflammation-induced DNA damage, which subsequently precipitates malignant transformation (Jena et al., 2012). Regarding the suppression of inflammation and OS, baicalin effectively alleviates UC in mouse models induced by 2,4,6-trinitrobenzene sulfonic acid (TNBS). This therapeutic effect is mediated by inhibition of the inhibitor of IKK/inhibitor of nuclear factor kappa-B (IκB)/NF-κB, which leads to the downregulation of pro-inflammatory cytokines, including IL-1β and TNF-α, as well as the modulation of apoptosis-related genes such as cysteinyl aspartate specific proteinase-9 (caspase-9) and B-cell lymphoma-2 (Bcl-2) (Shen et al., 2019a). Cui et al. reported that administration of baicalin to mice with TNBS-induced UC inhibits the TLR4/NF-κB signaling pathway, leading to a reduction in the production of downstream pro-inflammatory cytokines, including IL-1β, TNF-α, and IL-6, thereby achieving a therapeutic effect on UC (Cui et al., 2014). Similar findings were validated in experiments by Liang et al., where administration of baicalin and baicalein into UC rat models effectively decreased the serum levels of IL-1β, IL-17, and IL-6. Furthermore, these metabolites reduced the levels of OS products, specifically MDA and NO, while restoring the activity of the antioxidant enzyme SOD. Although both baicalin and its aglycone baicalein exhibit anti-inflammatory activity, their effects appear to be tissue-dependent. baicalin showed greater efficacy in colonic lesions, whereas. Baicalein exhibited relatively higher distribution to the small intestine and lungs. LC-MS/MS analysis further indicated that baicalin-related metabolites were differentially distributed in the large intestine, small intestine, lung, and serum, and baicalin showed greater efficacy in colonic lesions than baicalein. These findings suggest that baicalin may favor local intestinal exposure and may be more relevant than baicalein for targeting distal intestinal inflammation in UC. However, direct matching between this retained exposure and the concentrations required for NF-κB/MAPK inhibition or other cytoprotective effects remains insufficient (Liang et al., 2019).

Another study demonstrated that baicalin reshapes intestinal immune homeostasis through the microbiota-SCFAs-immune axis. Baicalin repaired physical and chemical barriers by upregulating ZO-1, occludin, and MUC2, enhanced antioxidant capacity by inhibiting ROS and MDA and increasing GSH and SOD activities, and corrected Th17/Treg imbalance. It also modulated the gut microbiota by reducing the F/B ratio and Proteobacteria abundance, thereby promoting the production of SCFAs, such as butyrate, and contributing to therapeutic effects in UC (Zhu et al., 2020). Furthermore, experiments in the *in vitro* simulation model suggested that baicalin initially decreases the expression of the transcription factor specificity protein 1 (SP1), resulting in a diminished binding affinity to the solute carrier family 6-member 14 (*SLC6A14*) gene promoter. The subsequent downregulation of *SLC6A14* expression inhibits TNF-α-induced apoptosis, inflammatory responses, and ferroptosis in the fetal human colon (FHC) epithelial cells. Collectively, these cytoprotective effects attenuate the onset and progression of UC (Sun H. et al., 2025).

Baicalin acts as an antitumor agent by inducing cell cycle arrest. Yang et al. reported that baicalin arrests cancer cells in the G1 phase and inhibits the TGF-β/Smad pathway. This inhibition reduces EMT and the expression of stemness proteins, thereby suppressing cell growth, migration, and proliferation while inducing apoptosis in colon tumor cells (Yang et al., 2020). Cai et al. demonstrated that baicalin significantly upregulates miR-139-3p expression in colon cancer cells. Mechanistically, miR-139-3p directly binds to the mRNA of cyclin-dependent kinase 16 (*CDK16*), thereby repressing its translation and protein expression. This inhibition induces cell cycle arrest and subsequently suppresses cancer cell proliferation, a conclusion validated by both gain- and loss-of-function assays (Cai et al., 2023). On the other hand, baicalin modulates key oncogenic signaling cascades. It suppresses the Wnt/β-catenin pathway, thereby downregulating downstream oncoproteins, including β-catenin and c-Myc. Concurrently, its antioxidant effects upregulate DEPP, which triggers apoptosis by activating the rat sarcoma viral oncogene homolog (Ras)/rapidly accelerated Fibrosarcoma viral oncogene homolog (Raf)/Mitogen-activated protein kinase (MEK)/ERK pathway (Wang et al., 2018; Jia et al., 2019). Furthermore, baicalin exerts indirect anti-tumor effects by regulating specific microRNAs, including miR-217, miR-10a, and miR-23a, which play pivotal roles in oncogene expression and tumor progression (Tao et al., 2018; Jia et al., 2019).

Ferroptosis is increasingly recognized as a pivotal tumor-suppressive mechanism and a promising therapeutic target for CRC (Lei et al., 2022; Yan H. et al., 2023). It plays a critical role in mitigating CRC progression by suppressing proliferation, metastasis, chemoresistance, and immune evasion (Lai et al., 2024). Mechanistically, baicalein exerts anti-tumor effects by inhibiting the Janus Kinase 2 (JAK2)/STAT3 signaling pathway. Specifically, baicalein targets JAK2 to block STAT3 phosphorylation, thereby repressing the transcription of GPX4. This downregulation of GPX4 subsequently induces ferroptosis in CRC cells (Lai et al., 2024).

### 3.3 Coptidis rhizoma (Huanglian)

*Coptidis Rhizoma* (Huanglian) consists of the dried rhizomes of *Coptis chinensis* Franch., *Coptis deltoidea* C. Y. Cheng et Hsiao, or *Coptis teeta* Wall. (Ranunculaceae). Metabolites isolated from Coptidis Rhizoma exhibit a broad spectrum of pharmacological activities, including antibacterial, antiviral, antifungal, antidiabetic, anticancer, and cardioprotective effects, as well as the regulation of gut microbiota (Wang et al., 2019). Coptis alkaloids such as berberine, epiberberine, palmatine, and berberrubine, are the representative bioactive metabolites of this botanical drug (Chen H. et al., 2017; Wang et al., 2019; Lu J. Z. et al., 2022).

Berberine is one of the active metabolites capable of treating UC. As an antimicrobial agent, berberine possesses anti-inflammatory, anticancer, hypoglycemic, and hypolipidemic properties (Luo X. F. et al., 2024). It is primarily used in the treatment of dysentery and intestinal infections, where it attenuates intestinal mucosal damage caused by pro-inflammatory cytokines (Yu et al., 2018). A Phase I clinical trial demonstrated that berberine significantly reduced Geboes scores in colonic tissues, though its effects on other tissues or blood biomarkers were limited. Co-administration enhanced mesalamine’s anti-inflammatory efficacy in colonic tissues (Xu L. et al., 2020). Meng et al. confirmed that immunity-related GTPase M family member 1 (IRGM1) is a direct anti-inflammatory target of berberine; by targeting IRGM1, berberine inhibits the downstream phosphoinositide 3-kinase (PI3K)/AKT/mammalian target of rapamycin (mTOR) pathway, reducing pro-inflammatory cytokines and increasing intestinal barrier proteins, thereby alleviating UC in mice (Meng et al., 2024). Li et al. verified that berberine mitigates UC-associated inflammation via the TLR4/NF-κB/hypoxia-inducible factor 1-alpha (HIF-1α) pathway, and in LPS-stimulated cells, it enhances structural integrity and reduces damage (Li et al., 2024a). Berberine also restores enteric glial cells (EGCs), maintains intestinal mucosal barrier homeostasis, and modulates immune cells through the “neuro-immune-epithelial” axis (Li H. et al., 2020). Moreover, a study by Jing et al. suggested that berberine possesses the capacity to restore the levels of microbial Trp metabolites, which are disrupted during UC development. These metabolites play a critical therapeutic role by activating AhR signaling, thereby improving intestinal barrier function and alleviating colonic inflammation (Jing et al., 2021). Epiberberine, another active metabolite of *Rhizoma Coptidis*, activates the intestinal farnesoid X receptor (FXR) to regulate enterohepatic circulation of bile acids. The restoration of intestinal bile acid homeostasis effectively downregulates expression of key inflammatory factors, such as NF-κB, thereby alleviating colonic inflammation (Chen M. et al., 2025). Furthermore, palmatine prevents UC by inhibiting inflammatory responses, OS, and iron overload, as well as suppressing the ferroptosis pathway (Ji et al., 2024). Berberrubine shows similar therapeutic effects to berberine at lower doses via dual mechanisms of inhibiting inflammation and restoring the physical barrier, suggesting its potential as a UC therapy (Yu et al., 2018).

An experimental study demonstrated that berberine remodels microbiota equilibrium and enhances the production of beneficial SCFAs, including butyrate, acetate, and propionate. This treatment also reduces endotoxin LPS levels while upregulating tight junction proteins (occludin and ZO-1) to restore the intestinal mucosal barrier. Furthermore, lowering the levels of LPS inhibit the TLR4/p-NF-κB p65/IL-6/p-STAT3 signaling axis, thereby blocking the progression from inflammation to carcinogenesis (Yan et al., 2022). Wang et al. showed that berberine remodels the gut microbiota and enhances intestinal amino acid and microbiota-derived SCFA metabolism. This process ultimately targets and inhibits the Wnt signaling pathway, which is closely associated with colorectal tumorigenesis, thereby suppressing tumor proliferation and promoting apoptosis (Wang M. et al., 2024). Meanwhile, the anti-cancer efficacy of berberine is highly dependent on its anti-inflammatory activity, with the core mechanism involving inhibiting polarization of the macrophages toward the pro-inflammatory (M1) phenotype and reducing pro-inflammatory cytokines, specifically IL-1β, IL-6, and TNF-α (Ling et al., 2023). Berberine suppresses the secretion of inflammatory factors by macrophages and inhibits the epidermal growth factor receptor (EGFR)/ERK signaling pathway. This blockade suppresses the abnormal proliferation of colonic epithelial cells and tumorigenesis, thereby exerting anti-inflammatory and anti-tumor effects. Collectively, these findings provide definitive molecular evidence for the application of anti-inflammatory agents in the treatment of intestinal tumors (Li et al., 2017). The RNA-binding protein Musashi-2 (MSI2) is required for the survival of CRC cells and represents a promising therapeutic target. Palmatine directly binds to the C-terminus of MSI2, thereby functionally antagonizing its oncogenic activity and effectively inhibiting CRC cell growth (Zhang et al., 2022). Furthermore, palmatine disrupts the interaction between the mitotic kinase Aurora Kinase A (AURKA) and Myosin-9 (MYH9), which hinders the nuclear localization of AURKA. This disruption leads to the downregulation of key regulatory proteins, ultimately inducing cell cycle arrest at the G2/M phase, thereby suppressing the malignant proliferation of CRC (Tang W. et al., 2024). Furthermore, berberrubine, a novel inhibitor of inosine monophosphate dehydrogenase isoform 2 (IMPDH2), suppresses CRC growth in both *in vitro* and *in vivo* models, thereby providing a fresh perspective on its potential therapeutic application in CRC (He et al., 2023).

### 3.4 Glycyrrhizae radix et rhizoma (Gancao)

*Glycyrrhizae Radix et Rhizoma*, derived from the dried roots and rhizomes of *Glycyrrhiza uralensis* Fisch., *Glycyrrhiza inflata* Bat., or *Glycyrrhiza glabra* L. (Leguminosae), is a renowned ancient herbal medicine that is most frequently prescribed in TCM (Yang et al., 2017). The botanical drug possesses a diverse chemical profile, including triterpenoid saponins, flavonoid glycosides, and free phenolic metabolites (Shang et al., 2022). Modern pharmacological studies indicate that its primary active metabolites are glycyrrhizic acid, glycyrrhizin, glycyrrhetinic acid, licoflavone B, and liquiritigenin, as well as alkaloids, polysaccharides, coumarins, and amino acids (Wu et al., 2025).

Glycyrrhizin, classified as a triterpenoid saponin, inhibits the function of high mobility group box 1 (HMGB1). This inhibition subsequently attenuates differentiation and proliferation of Th17 cells mediated by dendritic cells and macrophages, thereby reducing the production of inflammatory cytokines, such as IL-17, and mitigating colonic inflammation (Chen X. et al., 2017). Glycyrrhizic acid, a triterpenoid metabolite, exerts therapeutic effects in UC by synergistically interacting with piperine. This combination promotes polarization of macrophages from the M1 phenotype to the M2 anti-inflammatory phenotype via the mTOR/HIF-1α signaling axis, thereby correcting immune dysregulation (Wu et al., 2024). Licoflavone B, a flavonoid metabolite isolated and purified from the flavonoid fraction of licorice residues, directly blocks the MAPK signaling pathway to inhibit inflammatory responses in UC and remodels the gut microbiota. Concurrently, it suppresses colonic apoptosis and preserves the expression of occludin, claudin-1, and ZO-1, leading to maintenance of the integrity of the colonic barrier (Zhang J. et al., 2022). 18β-Glycyrrhetinic acid, another active metabolite extracted from *Radix et Rhizoma Glycyrrhizae*, functions through the Wnt/β-catenin signaling pathway. It enhances the expression of tight junction proteins occludin and ZO-1, while concurrently diminishing immune cell infiltration and alleviating aberrant immune attacks. Ultimately, these actions inhibit apoptosis of colonic epithelial cells and mitigate tissue injury, thereby achieving comprehensive therapeutic protection against UC (Peng et al., 2025).

In the context of cancer therapy, glycyrrhizic acid targets and inhibits the activity of peptidylarginine deiminase 4 (PAD4), thereby reducing the formation of neutrophil extracellular traps (NETs) and decreasing tumorigenicity. Concurrently, it restores the cytotoxicity of CD8^+^ T cells, enhancing their tumor-killing capacity. Together, these mechanisms exert a synergistic dual inhibitory effect on tumor progression (Chen Y. L. et al., 2025). Furthermore, isoliquiritigenin, a flavonoid metabolite, functions by activating the transient receptor potential vanilloid-1 (TRPV1) channel. This initiates calcium influx and intracellular calcium release, resulting in a significant elevation of cytosolic calcium concentrations. This calcium surge ultimately triggers the critical execution phase of apoptosis in cancer cells (Wang L. et al., 2025).

## 4 Therapeutic mechanisms of GQD in UC and potential adjunctive mechanisms in CRC

### 4.1 Mechanisms underlying the therapeutic effects of GQD on UC

**FIGURE 3:**
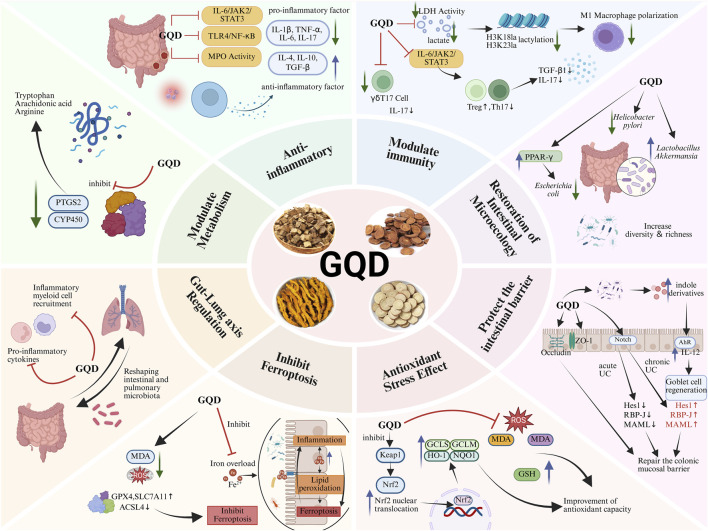
Schematic illustration of the therapeutic mechanisms of GQD in the treatment of UC.

GQD is extensively used in the clinical management of UC. Its therapeutic efficacy encompasses multiple facets, including anti-inflammation and immunomodulation, restoration of intestinal microecology, preservation of the intestinal barrier, alleviation of OS, inhibition of ferroptosis and metabolic reprogramming. Furthermore, GQD acts on the ‘gut-lung’ axis to treat UC-associated pneumonia and ameliorate extraintestinal manifestations. Collectively, these mechanisms exemplify the holistic advantage of GQD in exerting therapeutic effects through multi-pathway and multi-target strategies, which will be described in detail in the following sections. (Figure 3).

#### 4.1.1 Multi-pathway anti-inflammatory and immunomodulatory effects of GQD

In murine models of colitis, GQD significantly ameliorates body weight loss, colon shortening, and colonic inflammation (Hu et al., 2022). Neutrophil infiltration represents a hallmark pathological feature of UC. MPO, an indicator reflecting the extent of neutrophil infiltration, is significantly elevated in UC mice (Li N. et al., 2024). Zhao et al. reported that GQD targeted and inhibited the IL-6/JAK2/STAT3 signaling pathway, reduced the production of pro-inflammatory cytokines such as IL-1β, TNF-α, IL-6, IL-17, and TGF-β1, suppressed MPO activity, and alleviated inflammatory injury in colonic tissues (Zhao et al., 2021). Nuclear Factor kappa B (NF-κB) is well-recognized as a canonical inflammatory signaling pathway. Li et al. reported that GQD directly targeted the Toll-like Receptor 4 (TLR4)/NF-κB signaling pathway. By inhibiting the expression of TLR4, GQD blocked excessive downstream activation of NF-κB, thereby significantly reducing the expression of various pro-inflammatory cytokines such as TNF-α, IL-6, and IL-1β at the transcriptional level and effectively arresting the inflammatory cascade (Li et al., 2016). Moreover, a modified formulation of GQD downregulated the levels of pro-inflammatory cytokines (IL-1β, IL-17A, and IL-21) while concurrently upregulating anti-inflammatory cytokines (e.g., IL-4, IL-10, and TGF-β), thereby effectively mitigating intestinal inflammatory responses (Wang Y. et al., 2023).

Aberrant activation and polarization of macrophages is implicated in the immune dysregulation and sustained progression of inflammation in UC (Zhang et al., 2023). The abundance and dysfunction of macrophages is significantly increased during UC. Their hyperactivation—shifting from a protective inflammatory role to a destructive response—serves as a pivotal mechanism driving the transition of the intestinal mucosa from physiological inflammation to pathological injury (Na et al., 2019). Specifically, M1-polarized macrophages synergize with neutrophils and trigger Th1/Th17 immune responses to release inflammatory cytokines such as IFN-γ and IL-17, thereby establishing a pro-inflammatory vicious cycle. GQD reduces lactylation levels at specific histone sites (including H3K18la and H3K23la) by inhibiting lactate production and lactate dehydrogenase (LDH) activity. This modulation consequently suppresses M1 macrophage polarization, thereby alleviating intestinal inflammatory responses in UC (Xu Z. P. et al., 2024). The γδT cells constitute a unique population of innate-like T lymphocytes, comprising the Vδ1^+^ and Vγ9Vδ2 subsets. They serve as critical mediators in barrier surveillance, regulation of chronic inflammation, and formation of an immunosuppressive tumor microenvironment (Yang Y. et al., 2018). A modified formulation of GQD alleviates colitis by reducing the number of activated γδT 17 cells, thereby downregulating the expression of the key pro-inflammatory cytokine IL-17 (Ma et al., 2023). Furthermore, by regulating the key IL-6/JAK2/STAT3 signaling pathway, GQD ameliorates the infiltration balance between Tregs and Th17 in colonic tissues. It decreases the frequencies of Treg and Th17 cells in Peyer’s patches and the spleen, reduces the expression of inflammatory cytokines (TGF-β1 and IL-17), and alleviates DSS-induced UC (Zhao et al., 2021).

#### 4.1.2 Restoration of intestinal microecology by GQD

GQD enhances the diversity and abundance of beneficial gut bacteria and maintains intestinal microecological homeostasis, which is pivotal for the restoration of the intestinal mucosal barrier and the suppression of intestinal inflammation (Yang et al., 2025a). Hu et al. reported that GQD exerts its therapeutic effects by remodeling the gut microbiota. Specifically, it inhibits the overgrowth of opportunistic pathogens such as *Helicobacter*, while concurrently promoting the proliferation of beneficial genera like *Lactobacillus* and *Akkermansia*. Notably, Fecal Microbiota Transplantation experiments confirmed that the GQD-regulated gut microbiota are sufficient to independently mediate these therapeutic effects. This efficacy is intimately associated with the modulation of Th2/Th1 and Treg/Th17 immune homeostasis, inhibition of the NLRP3 inflammasome activation, and enhancement of the colonic barrier function (Hu Y. et al., 2024).

The nuclear receptor PPARγ is a member of the nuclear receptor superfamily and plays a pivotal role in regulating intestinal homeostasis. PPARγ synthesis is diminished in epithelial cells of mice with UC and DSS-induced colitis (Bouguen et al., 2015; Klepsch et al., 2019; Cevallos et al., 2021). Hu et al. corroborated this finding and demonstrated that GQD exerts therapeutic effects against UC by activating the PPARγ signaling pathway. This regulation inhibits the expression of iNOS and the generation of nitrates in the colonic epithelium. Consequently, it effectively suppresses the abnormal proliferation of nitrate-respiration-dependent Enterobacteriaceae (such as *Escherichia coli*), while concurrently reducing intestinal LPS levels and alleviating inflammatory responses (Hu et al., 2022).

#### 4.1.3 Protective effects of GQD on the intestinal barrier

The intestinal mucus barrier, composed primarily of mucins secreted by goblet cells, acts as the host’s first line of defense, shielding the intestinal epithelium from invasion. Hu et al. reported that GQD administration ameliorates intestinal barrier dysfunction by upregulating the expression of intestinal epithelial tight junction proteins (occludin and ZO-1), reducing intestinal permeability, and repairing the colonic mucus barrier (Hu Y. et al., 2024). GQD restores the intestinal barrier via the ‘microbiota-metabolism-immune’ axis. Mechanistically, GQD remodels the gut microbiota composition and promotes Trp metabolism to generate indole derivatives, which subsequently activate the AhR signaling pathway and upregulate IL-22 levels. Ultimately, this cascade alleviates UC by restoring intestinal barrier function through goblet cell regeneration and tight junction repair (Wang et al., 2023b).

Crypt Base Columnar (CBC) stem cells sustain regeneration and renewal of the intestinal tissue (Rodríguez-Colman et al., 2017; Capdevila et al., 2024). The Notch signaling pathway directly targets CBC cells to sustain stem cell activity and plays a pivotal role in dictating cell fate determination between absorptive and secretory lineages in the intestinal epithelium (VanDussen et al., 2012). GQD exerts a dynamic, context-dependent regulatory effect. In acute UC models, GQD suppresses hyperactive Notch signaling by downregulating key effector proteins such as hes family bHLH transcription factor 1(Hes1), recombination signal binding protein-J (RBP-J), and mastermind-like (MAML), thereby facilitating goblet cell differentiation and mucosal repair. Conversely, in the chronic phase of UC, GQD enhances Notch signaling activity by upregulating these proteins. This shift prevents excessive goblet cell differentiation and promotes CBC stem cell proliferation, thereby accelerating epithelial regeneration. Collectively, these findings confirm that the therapeutic efficacy of GQD is intrinsically linked to its bidirectional modulation of the Notch signaling pathway, which restores colonic mucosal homeostasis (Zhao et al., 2020).

#### 4.1.4 Antioxidant effects of GQD

The pathogenesis of UC is closely associated with OS. The Nrf2 signaling pathway is a pivotal intrinsic mechanism by which the host counteracts oxidative injury and promotes mucosal repair. Consequently, the functional status of the Nrf2 pathway may influence the pathological progression and recovery potential of UC (Arisawa et al., 2007; Arisawa et al., 2008; Eftekharzadeh et al., 2010; Cuadrado et al., 2019). Lin et al. demonstrated that GQD activates Keap1/Nrf2/ARE signaling in DSS-induced UC rats and TNF-α-stimulated Caco-2 cells, leading to upregulation of NAD(P)H:quinone oxidoreductase 1 (NQO1), HO-1, Glutamate-Cysteine Ligase Catalytic Subunit (GCLC), and Glutamate-Cysteine Ligase Modifier Subunit (GCLM) and restoration of redox homeostasis (Lin et al., 2022). However, this study mainly focused on inflammatory and oxidative stress models and did not evaluate whether sustained Nrf2 activation may be associated with potential pro-carcinogenic risks. In addition, as discussed above, Nrf2 exerts context-dependent dual effects, including both protective and potential pro-tumorigenic roles. Therefore, whether long-term GQD-mediated Nrf2 activation may increase the risk of CRC development remains uncertain and requires further rigorous investigation. Concurrently, GQD effectively mitigates OS injury in the colonic tissues. Specifically, this is characterized by reduced levels of the OS marker MDA and inflammatory cell infiltration indicator MPO, as well as elevated levels of the critical endogenous antioxidant GSH, thereby restoring redox homeostasis (Li et al., 2016).

Wang et al. demonstrated that a modified formulation of GQD ameliorates damage in both DSS-induced UC mice and a TNF-α-induced Caco-2 cell monolayer intestinal barrier model, as indicated by reduced levels of MDA and upregulated expression of key antioxidant enzymes such as SOD, CAT, and GSH, thereby restoring redox homeostasis (Wang Y. et al., 2023).

#### 4.1.5 Regulation of UC by GQD through cell death and metabolic pathways

Ferroptosis is a distinct form of programmed cell death. Chen et al. demonstrated that suppressing ferroptosis effectively ameliorates DSS-induced UC by suppressing the Nrf2/HO-1 signaling pathway (Chen et al., 2020). Consequently, ferroptosis serves as a potential therapeutic target for the treatment of UC. GQD alleviates UC by inhibiting ferroptosis in IECs. Mechanistically, in both *in vivo* and *in vitro* models, GQD significantly ameliorates key ferroptosis markers. This effect is specifically characterized by a reduction in iron overload, MDA, and mitochondrial reactive oxygen species (mtROS), as well as modulation of ferroptosis-related protein expression as demonstrated by upregulation of GPX4 and solute carrier family 7 member 11(SLC7A11) and downregulation of acyl-coA synthetase long chain family member 4 (ACSL4). Furthermore, *in vivo* investigations demonstrate that GQD inhibits Keap1 to promote Nrf2 nuclear translocation and activate its downstream signaling pathway. This activation subsequently upregulates the expression of multiple antioxidant proteins, including HO-1 and NQO1, and key glutathione synthesis enzymes, GCLC and GCLM, ultimately enhancing the host antioxidant capacity to alleviate the disease (Lin et al., 2022). Bioactive metabolites within GQD, including puerarin, berberine, and baicalin, possess distinct anti-ferroptotic properties. Collectively, these actions attenuate oxidative stress-induced injury to the intestinal epithelium and repair epithelial barrier function, thereby exerting therapeutic effects against UC (Wang et al., 2023c).

In terms of metabolism, GQD significantly reverses dysregulated metabolites in UC models, specifically regulating the Trp, arginine, and proline metabolism pathways, thereby correcting systemic metabolic dysregulation (Yang et al., 2025a). Integrated metabolomics and network pharmacology results have demonstrated that GQD exerts therapeutic effects against UC by systematically regulating arachidonic acid metabolism and Trp metabolism. The core mechanism is characterized by the ability of bioactive metabolites within GQD to directly inhibit the protein expression of key metabolic enzymes, such as prostaglandin-endoperoxide synthase 2 (PTGS2) and cytochrome P450 (CYP450), thereby correcting UC-associated metabolic dysregulation. This provides solid evidence at the metabolic level for the efficacy of GQD (Zhang M. et al., 2025). A study showed that GQD may also restore amino acid and purine metabolic homeostasis by regulating gut microbiota-associated metabolic functions, thereby further suppressing pro-inflammatory mediators such as TNF-α, IFN-γ, and IL-17. In addition, this study found that GQD could modulate colonic γδT cell responses and reduce IFN-γ^+^γδT- and IL-17^+^γδT-associated pro-inflammatory responses, suggesting that its metabolic regulatory effects may be interconnected with remodeling of the mucosal immune microenvironment (Shao et al., 2026). Taken together, these findings indicate that the metabolic effects of GQD should be understood as an integrated regulatory process involving gut microbiota-derived metabolic functions, host metabolic homeostasis, and mucosal immune remodeling, rather than as isolated changes in individual metabolites.

#### 4.1.6 Potential role of GQD in extraintestinal manifestations: the gut–lung axis

Concurrently, GQD exerts a bidirectional regulatory role within the ‘gut-lung axis.’ GQD plays a central role in treating UC and related pulmonary inflammation by mediating the bidirectional crosstalk between the gut and lung via the ‘microbiota-immune’ axis. Immunologically, GQD effectively inhibits recruitment of inflammatory myeloid cells such as neutrophils and macrophages, and release of key pro-inflammatory cytokines, such as TNF-α, IL-1β, and IL-6. GQD also remodels the microbiota composition in both the gut and lungs by regulating specific key bacterial genera. Crucially, significant correlations exist between these GQD-regulated microbes and both inflammatory markers and immune cells. This reveals a multi-target therapeutic mechanism whereby GQD synergistically regulates microbial communities and host immune responses along the ‘gut-lung axis,’ thereby achieving anti-inflammatory effects and microecological balance across multiple organs (Li et al., 2022).

### 4.2 Potential adjunctive mechanisms of GQD in CRC

Although current evidence for the direct treatment of CRC with GQD remains limited, particularly in terms of pathway-specific mechanistic research, available studies suggest that GQD may still have therapeutic relevance in this setting. Rather than acting as a primary anticancer agent, GQD appears to exert its effects mainly through adjuvant mechanisms, especially by enhancing the efficacy of conventional antitumor therapies and alleviating treatment-related adverse reactions. Therefore, this section summarizes the potential role of GQD in CRC from these two perspectives.

#### 4.2.1 Synergistic effects of GQD in combination with conventional CRC therapies

The development of immunotherapeutic agents has significantly improved the overall survival and progression-free survival of many cancer patients. Targeting the programmed cell death protein 1 (PD-1), an immune checkpoint receptor, enhances immune surveillance and the elimination of cancer cells in CRC (Lin et al., 2023). However, majority of clinical CRC cases are classified as microsatellite stable (MSS), a subtype that typically exhibits a poor response to immunotherapies such as PD-1 inhibitors. Consequently, combination regimens are ideal to overcome this limitation. GQD inhibits tumor growth when administered in combination with anti-PD-1 antibodies and exerts synergistic effects. Mechanistically, GQD remodels the gut microbiota and regulates metabolic pathways, specifically glycerophospholipid and sphingolipid metabolism. This enhances the infiltration and function of CD8^+^ T cells within the TME. Furthermore, combination therapy promotes expression of IFN-γ and IL-2 while downregulating PD-1 levels. This process effectively reverses T cell exhaustion and reshapes the TME to synergistically potentiate antitumor immunity, thereby providing a promising therapeutic strategy for MSS-type CRC (Lv et al., 2019).

TLR4 represents an important pattern recognition receptor for intestinal immunity, and is frequently overexpressed in colonic epithelial cells under inflammatory conditions. Emerging evidence suggests that TLR4 serves as a biomarker and promotes CRC progression (Fukata et al., 2011). GQD effectively suppresses the phosphorylation and nuclear translocation of NF-κB and IRF3 by inhibiting the TLR4/myeloid differentiation primary response protein 88 (MyD88) signaling pathway. This downregulates the expression of key pro-inflammatory cytokines, such as IL-1β, IL-6, and TNF-α, thereby exerting its antitumor effects (Xu Y. et al., 2024).

The combination of GQD extract and irinotecan (CPT-11) may exert synergistic anticancer effects and potentially reduce treatment-related toxicity in CRC models. In terms of immune modulation, GQD appears to reshape the tumor immune microenvironment by reducing the population of Tregs while enhancing the infiltration of CD4^+^ and CD8^+^ T cells. Concurrently, GQD inhibits glucose uptake in CT-26 cells and decreases cell viability under anaerobic glycolytic conditions, suggesting disruption of the Warburg effect (Yang et al., 2025b). Recent multi-omics evidence further suggests that GQD may remodel the immune-metabolic microenvironment in UC-associated CRC. When combined with capecitabine plus oxaliplatin (XELOX), GQD reduced inflammatory and oxidative stress-related factors, improved immune-related indicators, reshaped gut microbiota composition, and restored metabolic disturbances. Mechanistically, this effect was associated with regulation of unsaturated fatty acid metabolism and downregulation of arachidonate 15-lipoxygenase (ALOX15), cytochrome P450 family 1 subfamily B member 1 (CYP1B1), and PTGS2, suggesting that GQD may enhance chemotherapy efficacy by coordinating immune modulation and metabolic reprogramming (Tang Q. et al., 2024). These studies suggest that GQD may influence CRC by modulating the tumor immune microenvironment and reprogramming cancer cell metabolism, indicating a coordinated effect on immune and metabolic pathways.

**FIGURE 4:**
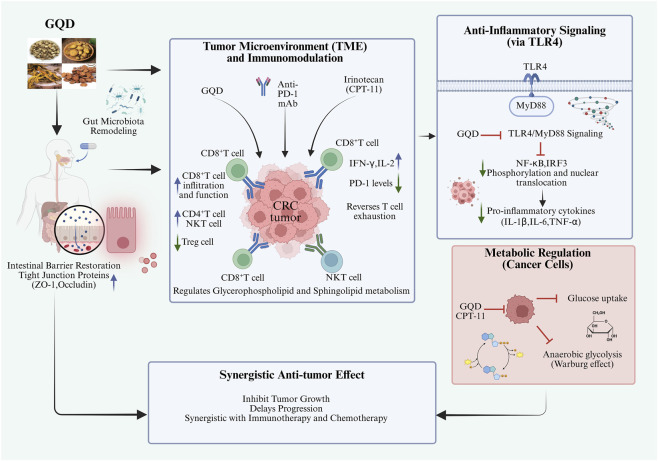
Schematic illustration of the potential adjunctive mechanisms of GQD in CRC.

Another randomized controlled trial (RCT) involving CRC patients demonstrated that GQD administration increased the numbers of CD4^+^ T cells while concurrently upregulating Natural Killer T (NKT) cells, thereby enabling these populations to synergistically exert antitumor effects. Furthermore, GQD treatment significantly reduced TNF-α levels, thereby alleviating inflammation in CRC patients. It also restored intestinal barrier function by enhancing the expression of tight junction proteins ZO-1 and occludin. GQD delays CRC progression by remodeling the gut microbiota (Li Y. et al., 2020) (see Figure 4).

#### 4.2.2 Role of GQD in alleviating treatment-related adverse effects in CRC patients

Conventional therapeutic agents used in cancer treatment are characterized by their non-specific tissue distribution and potential to induce toxicity, which can result in significant damage to non-malignant tissues and cells. The clinical application of these antitumor agents is frequently limited by adverse reactions such as myelosuppression, hepatic dysfunction, gastrointestinal toxicity, and drug resistance, which compromises their therapeutic efficacy (Cheng et al., 2014; Qi et al., 2015; Wu et al., 2019; Liu et al., 2024; Wang C. et al., 2025). However, GQD alleviates these adverse effects and functions synergistically with chemoradiotherapy in the treatment of CRC.

CPT-11, a water-soluble semisynthetic analog of camptothecin (CPT), is widely used in the treatment of various malignancies due to its potent anticancer efficacy. However, its clinical application is significantly hindered by potential dose-limiting toxicities, especially intestinal toxicity (specifically, delayed-onset diarrhea) (Kon et al., 2018). An experimental study involving a mouse model of CPT-11-induced diarrhea demonstrated that GQD alleviated intestinal toxicity by reducing colonic inflammation levels, ameliorating OS, and protecting intestinal barrier function. GQD exerted a robust synergistic tumor-suppressive effect without compromising the anticancer efficacy of CPT-11 (Wu et al., 2019). In another study, GQD effectively mitigated intestinal toxicity triggered by CPT-11 by regulating the cytokine profile and promoting the polarization of colonic macrophages from the pro-inflammatory M1 phenotype to the anti-inflammatory and reparative M2 phenotype (Yang et al., 2025b).

YTH N6-methyladenosine RNA Binding Protein F1 (YTHDF1), an N6-methyladenosine (m6A) reader protein, functions primarily by specifically recognizing and binding to m6A modifications prevalent on messenger RNA (mRNA). YTHDF1 expression in CRC facilitates tumor progression by suppressing T cell function via activation of the m6A-p65-CXCL1 axis (Bao et al., 2023). The overexpression of YTHDF1 upregulates the expression of glutaminase 1 (GLS1), thereby inducing resistance to oxaliplatin in CRC. When co-administered with oxaliplatin, GQD demonstrates a favorable synergistic effect. It effectively reverses drug resistance by suppressing the expression of both YTHDF1 and GLS1, as well as inhibiting GLS1 activity (Lin et al., 2024).

### 4.3 GQD-associated hierarchical regulatory network

Overall, the effects of GQD should not be understood as the isolated regulation of a single molecular marker, but may instead reflect an integrated intervention in a multilayered pathological network. This network can be summarized into four levels: upstream pathological triggers, core signaling hubs, downstream cellular responses, and disease-level outcomes. At the upstream level, GQD may regulate pathological factors such as gut microbiota dysbiosis, bacterial and metabolite translocation, inflammatory burden, immune microenvironment disorder, oxidative stress, metabolic disturbance, and abnormal epithelial cell death, thereby weakening the driving forces that sustain chronic intestinal inflammation and promote the formation of a pro-carcinogenic microenvironment. These upstream events further converge on multiple core signaling hubs, including TLR4/NF-κB, IL-6/JAK2/STAT3, the NLRP3 inflammasome, Keap1/Nrf2/ARE, AhR/IL-22, Notch, PPARγ, and the gut microbiota–metabolite signaling axis. By regulating these hubs, GQD may further influence downstream effects, including macrophage polarization, Th17/Treg balance, γδT-cell inflammatory activity, cytokine cascades, tight junction reconstruction, mucus production, antioxidant defense, and ferroptosis-related epithelial injury.

From a biological perspective, this hierarchical network suggests that the potential effects of GQD on inflammation alleviation and barrier repair may not rely solely on a single pathway, but may arise from the joint regulation of multiple pathological feedback loops. During the inflammatory stage before cancer formation, inhibition of inflammatory hubs by GQD may reduce cytokine-driven epithelial injury and interrupt macrophage- and Th17-related inflammatory amplification. Restoration of the mucus layer and tight junction barrier may limit bacterial translocation and recurrent immune activation. Regulation of oxidative stress, metabolism, and ferroptosis may further reduce lipid peroxidation, epithelial cell loss, and oxidative DNA damage, thereby weakening the colitis-associated pro-carcinogenic microenvironment. In diagnosed CRC, current evidence appears to place greater emphasis on the adjunctive therapeutic role of GQD, particularly through gut microbiota remodeling, regulation of the tumor immune microenvironment, improvement of treatment tolerance, and modulation of therapy resistance, rather than direct cytotoxic anticancer activity. Future studies should further distinguish the effects of GQD in inflammatory, precancerous, and established tumor settings, using appropriate experimental models to clarify whether its mechanisms and therapeutic relevance differ across UC, CAC, and CRC.

## 5 Current limitations and future perspectives

Although evidence regarding GQD and its representative bioactive constituents has continued to accumulate, the current evidence base remains largely preclinical and is still insufficient to support definitive clinical translation. Several major limitations continue to restrict cross-study comparability and clinical interpretation, including insufficient high-quality clinical evidence, incomplete formula standardization, heterogeneity in experimental models and dosing regimens, insufficiently characterized PK–PD and bioavailability profiles, and limited causal validation of the proposed mechanisms.

### 5.1 Strengthening clinical validation through standardized trial design

The most important evidence gap remains the lack of high-quality clinical studies. Future clinical investigations should adopt multicenter, randomized, double-blind, controlled trial designs with clearly defined patient populations, standardized interventions, and pre-specified outcome measures. For UC, clinically meaningful endpoints may include clinical response and remission rates, Mayo score, endoscopic mucosal healing, histological improvement, relapse rate, inflammatory biomarkers, and quality-of-life indicators (Kedia et al., 2022; Yin et al., 2022; Khazdouz et al., 2023; Estevinho et al., 2024; Gisbert et al., 2024). For CRC, unless stronger evidence becomes available, GQD should not be evaluated as an independent cytotoxic anticancer therapy. To improve transparency and reproducibility, clinical trials of GQD should follow reporting frameworks such as CONSORT-CHM Formula 2017 (Wang J. et al., 2024). Such standardized reporting would help determine whether the observed clinical effects can be reliably attributed to GQD and would facilitate comparisons across future studies.

### 5.2 Establishing formula standardization and quality-marker-based control

A major challenge in the translational development of herbal formulas is batch-to-batch variability. For GQD, future studies should establish a standardized quality-control system that integrates botanical authentication, chemical fingerprinting, multi-component quantitative analysis, and bioactivity-related quality markers (Chen et al., 2023; Yan et al., 2025). Importantly, quality control should not rely solely on the abundance of a few marker compounds. Establishing quality markers associated with intestinal exposure, pharmacological activity, and disease-relevant endpoints would be a more effective strategy (Lu X. et al., 2022; Seo and Shin, 2022; Yang et al., 2024). For example, candidate quality markers could be screened by integrating chemical analysis, network pharmacology, bioactivity assays, and other approaches. These markers could then be validated by determining whether different batches of GQD with similar chemical fingerprints exert consistent effects on disease-related markers.

### 5.3 Optimizing oral bioavailability and colon-targeted exposure

Because GQD is traditionally administered orally, future studies should pay greater attention to its PK–PD characteristics, bioavailability, tissue distribution, and colon-targeted exposure. The systemic bioavailability of different bioactive constituents or metabolites may vary (Lu J. Z. et al., 2022). More importantly, interspecies metabolic differences should also be taken into consideration. Recent comparative evidence on hepatic metabolism further suggests that GQD constituents may exhibit species-dependent metabolic patterns between rats and humans, indicating that bioavailability optimization and PK–PD interpretation should consider not only intestinal absorption, but also host metabolic transformation and target-tissue exposure (Wang et al., 2026). Therefore, future studies should not be limited to quantifying plasma concentrations, but should also simultaneously assess the levels of parent compounds and their metabolites in intestinal tissues and metabolic organs such as the liver. In addition, interspecies differences should be incorporated into PK–PD interpretation to more accurately determine the *in vivo* exposure characteristics and clinical translational relevance of GQD.

New drug delivery systems also represent an important direction in the translational research of traditional Chinese medicine formulas. Strategies such as nanoparticles, microsphere-based delivery systems, micelles, pH-responsive delivery systems, and exosome-like vesicles have continued to emerge, highlighting the prospect of diversified drug administration approaches (Chang et al., 2023; Tie et al., 2023; Li et al., 2025; Ning et al., 2025; Wang M. et al., 2025). Recent evidence suggests that natural nanoparticles derived from GQD may function as an intrinsic oral delivery system. These nanoparticles are rich in proteins and polysaccharides, exhibit a certain degree of gastrointestinal stability, and may enhance the colonic absorption and tissue distribution of multiple active constituents (Mu et al., 2025). Future studies are still needed to further explore the feasibility of diversified delivery approaches for GQD, with the aim of improving drug stability, solubility, intestinal retention time, or local delivery efficiency.

### 5.4 Improving experimental reproducibility through model, dose, and endpoint harmonization

At present, heterogeneity in disease models, doses, administration routes, and experimental endpoints limits comparability across studies (Bramhall et al., 2015). Future preclinical studies should adopt more standardized and disease-relevant experimental designs. Researchers should select disease models according to the specific pathological questions being addressed, and ideally, key findings should be validated across multiple models (Baydi et al., 2021). Dose selection should also be more transparent and clinically interpretable. Dose selection should also be more transparent and clinically interpretable. Due to the use of single-dose regimens, varied administration routes, and heterogeneous disease models in many studies, current experimental evidence remains insufficient to define the minimum effective dose of GQD-related metabolites. Therefore, dose-gradient designs should be combined with human equivalent dose calculation and tissue exposure assessment to distinguish clinically relevant pharmacological effects from potentially suprapharmacological responses. Experimental endpoints should also be harmonized, including clinical disease activity, histological scores, epithelial barrier integrity and tumor burden when appropriate. Such harmonization would improve the comparability of future studies and reduce the risk of overinterpreting isolated findings.

### 5.5 Moving from associative mechanisms to causal validation

Although many studies have reported changes in inflammatory mediators, oxidative stress markers, gut microbiota composition, barrier-related proteins, metabolic pathways, and cell death-related molecules after GQD treatment, most current mechanistic evidence remains largely associative. Therefore, future studies should adopt causal validation strategies to determine which pathways are necessary or sufficient for the observed effects. For example, pathway inhibitors, gene knockdown or knockout models, and organoid-based validation could be used to examine whether specific mechanisms mediate the protective effects of GQD (Schulte et al., 2019; Graham and Xavier, 2020).

Overall, future research on GQD should shift from descriptive efficacy and mechanistic reporting toward standardized, causally validated, and clinically translatable investigation. Establishing reproducible formula standardization, clarifying pharmacokinetic and colonic exposure characteristics, harmonizing experimental models and endpoints, validating causal mechanisms, and conducting rigorously designed clinical trials will be essential for determining the translational value of GQD in UC and its potential chemopreventive or adjunctive role in inflammation-associated colorectal carcinogenesis.

## 6 Conclusion

GQD, as a classical traditional Chinese medicine formula, has attracted considerable attention because of its potential roles in UC and CRC. This review focuses on the inflammation-to-cancer transition and systematically summarizes recent advances regarding GQD and its representative bioactive constituents from the perspectives of chronic intestinal inflammation, gut microbiota dysbiosis, epithelial and mucus barrier disruption, oxidative stress, metabolic disturbance, dysregulated cell death, and tumor microenvironment remodeling. Current studies suggest that the mechanisms of GQD may involve a multilayered regulatory network. GQD may collectively promote inflammation alleviation and mucosal repair by suppressing pro-inflammatory signaling pathways, modulating immune cell imbalance, restoring gut microbiota homeostasis, protecting epithelial barrier integrity, reducing oxidative injury, regulating metabolic abnormalities, and inhibiting ferroptosis-related epithelial injury, thereby potentially influencing the pathological process of UC progression toward CRC.

Importantly, the role of GQD in CRC should be interpreted cautiously. Current evidence does not support the use of GQD as a first-line cytotoxic anticancer therapy for established CRC. Instead, existing findings suggest that GQD may be more appropriately considered as an adjunctive therapeutic agent, particularly in relation to gut microbiota remodeling, regulation of the tumor immune microenvironment, improvement of treatment tolerance, alleviation of treatment-related intestinal toxicity, and modulation of therapy resistance. This distinction is essential to avoid overinterpreting the consistency of preclinical mechanisms as confirmed clinical efficacy.

The mechanistic framework summarized in this review provides a reasonable explanation for how GQD may intervene in the inflammation-to-cancer transition from UC to CRC. However, this interpretation is mainly based on preclinical studies, including animal experiments, cell models, and limited clinical observations. Limitations such as insufficient clinical evidence, incomplete formula standardization, the need for further optimization of bioavailability, heterogeneity in experimental methods, and insufficient causal validation of the proposed mechanisms indicate that the translational value of GQD still requires verification through rigorously designed clinical studies. Future research should focus on strengthening clinical validation through standardized trial design; establishing formula standardization and quality-marker-based control; optimizing oral bioavailability and colon-targeted exposure; improving experimental reproducibility through harmonization of models, doses, and endpoints; and moving from associative mechanisms to causal validation. Nevertheless, GQD may participate in the regulation of UC-related inflammation and intestinal barrier injury through multiple mechanistic pathways, and may also have potential adjunctive value in CRC-related treatment, highlighting its broad application prospects.

## Glossary

TNBS: 2,4,6-trinitrobenzene sulfonic acid
ACSL4: Acyl-CoA Synthetase Long Chain Family Member 4
APC: Adenomatous Polyposis Coli
AIEC: Adherent-invasive *Escherichia coli*
ARE: Antioxidant response element
Arg-1: Arginase-1
AhR: Aryl hydrocarbon receptor
AURKA: Aurora Kinase A
AOM/DSS: Azoxymethane/Dextran sulfate sodium
Bcl-2: B-cell lymphoma-2
CPT: Camptothecin
CAT: Catalase
CIN: Chromosomal Instability
CDI: *Clostridium difficile infection*
CAC: Colitis-associated Cancer
CRC: Colorectal Cancer
CMS2: Consensus Molecular Subtype 2
CD: Crohn’s disease
CBC: Crypt Base Columnar
CDK16: Cyclin-Dependent Kinase 16
COX-2: Cyclooxygenase-2
caspase-9: Cysteinyl Aspartate Specific Proteinase-9
CYP450: Cytochrome P450
CMV: Cytomegalovirus
DSS: Dextran Sulfate Sodium
EGCs: Enteric Glial Cells
EGFR: Epidermal Growth Factor Receptor
EMT: Epithelial-Mesenchymal Transition
EBV: Epstein–Barr virus
ERK: Extracellular Signal-Regulated Kinase
FXR: Farnesoid X Receptor
FHC: Fetal Human Colon
F/B: *Firmicutes/Bacteroidetes*
Foxp3: Forkhead Box P3
GQD: Gegen Qinlian Decoction
Gli1: Glioma-associated Oncogene Homolog 1
GCLC: Glutamate-Cysteine Ligase Catalytic Subunit
GCLM: Glutamate-Cysteine Ligase Modifier Subunit
GLS1: Glutaminase 1
GSH: Glutathione
GPx: Glutathione Peroxidase
GPX4: Glutathione Peroxidase 4
Hh: Hedgehog
Th: Helper T
HO-1: Heme Oxygenase-1
Hes1: Hes family bHLH transcription factor 1
HMGB1: High Mobility Group Box 1
HIV: Human Immunodeficiency Virus
HLA: Human Leukocyte Antigen
H2O2: Hydrogen peroxide
HIF-1α: Hypoxia-Inducible Factor 1-Alpha
IRGM1: Immunity-related GTPase M family member 1
IAA: Indole-3-acetic acid
ILA: Indole-3-lactic acid
IPA: Indole-3-propionic acid
iNOS: inducible Nitric Oxide Synthase
IBD: Inflammatory bowel disease
IκB: inhibitor of nuclear factor kappa-B
IMPDH2: Inosine Monophosphate Dehydrogenase Isoform 2
IRF4: Interferon Regulatory Factor 4
IRF5: Interferon Regulatory Factor 5
IFN-γ: Interferon-γ
IL-10: Interleukin-10
IL-13: Interleukin-13
IL-17: Interleukin-17
IL-17A: Interleukin-17A
IL-1β: Interleukin-1β
IL-2: Interleukin-2
IL-21: Interleukin-21
IL-22: Interleukin-22
IL-23: Interleukin-23
IL-4: Interleukin-4
IL-5: Interleukin-5
IL-6: Interleukin-6
IECs: Intestinal epithelial cells
CPT-11: Irinotecan
JAK2: Janus Kinase 2
Keap1: Kelch-like ECH-associated protein 1
LDH: Lactate Dehydrogenase
LPS: Lipopolysaccharide
MHC: Major Histocompatibility Complex
MDA: Malondialdehyde
mTOR: Mammalian Target of Rapamycin
MAML: Mastermind-like
MMPs: Matrix Metalloproteinases
mRNA: Messenger RNA
MMP-9: Metalloproteinase-9
MSS: Microsatellite Stable
MAPK: Mitogen-activated Protein Kinase
MEK: Mitogen-activated protein kinase
MCP-1: Monocyte Chemoattractant Protein-1
Muc2: Mucin 2
MSI2: Musashi-2
MyD88: Myeloid Differentiation Primary Response Protein 88
MPO: Myeloperoxidase
MYH9: Myosin-9
NQO1: NAD(P)H:quinone oxidoreductase 1
NKT: Natural Killer T
NETs: Neutrophil extracellular traps
NO: Nitric Oxide
NLRP3: NOD-like receptor family pyrin domain containing 3
NAFLD: Non-alcoholic fatty liver disease
Nrf2: Nuclear factor erythroid 2-related factor 2
NF-κB: Nuclear Factor kappa B
IKK: nuclear factor kappa-B kinase
OS: Oxidative Stress
PAD4: Peptidylarginine deiminase 4
PPARγ: Peroxisome Proliferator-activated Receptor-γ
PK–PD: Pharmacokinetics–Pharmacodynamics
PI3K: Phosphoinositide 3-kinase
p-IκB-α: Phosphorylated Inhibitor of NF-κB alpha
p-IKK: Phosphorylated IκB Kinase
p65-NF-kappaB: Phosphorylated p65 Nuclear Factor kappa-B
PID: Primary Immunodeficiencies
PD-1: Programmed Cell Death Protein 1
PTGS2: Prostaglandin-Endoperoxide Synthase 2
AKT: Protein kinase B
RCT: Randomized Controlled Trial
Raf: Rapidly accelerated Fibrosarcoma viral oncogene homolog
Ras: Rat sarcoma viral oncogene homolog
ROS: Reactive Oxygen Species
RBP-J: Recombination Signal Binding Protein-J
Tregs: Regulatory T cells
SCFAs: Short-chain fatty acids
STAT3: Signal Transducer and Activator of Transcription 3
SNPs: single nucleotide polymorphisms
SLC6A14: Solute Carrier Family 6-member 14
SLC7A11: Solute Carrier Family 7 Member 11
SP1: Specificity Protein 1
SOD: Superoxide Dismutase
Th17: T helper 17
TJs: tight junctions
TLR4: Toll-like receptor 4
TCM: Traditional Chinese Medicine
TGF-β: Transforming growth factor-β
TGF-β1: Transforming Growth Factor-β1
TRPV1: Transient Receptor Potential Vanilloid-1
Trp: Tryptophan
TIME: Tumor Immune Microenvironment
TME: Tumor Microenvironment
TNF-α: Tumor Necrosis Factor-α
UC: Ulcerative Colitis
VEO-IBD: Very early-onset Inflammatory Bowel Disease
YTHDF1: YTH N6-methyladenosine RNA Binding Protein F1
ZO-1: Zonula occludens-1
