# Supplementary material for: Gegen Qinlian Decoction, a classic traditional Chinese medicine formula: a potential therapeutic strategy for ulcerative colitis and colorectal cancer
Source: Front Pharmacol. 2026 Jul 15;17:1868875. doi: 10.3389/fphar.2026.1868875 (PMC13415777; doi:10.3389/fphar.2026.1868875)
Supplement: Supplementary file 1 [file Table1.docx]

Table S1. Representative bioactive metabolites of GQD and their pharmacological activities in UC.

| Botanical Drug & Validated Taxon  (Family) | Metabolite | Experimental Model  (*In vivo* / *In vitro*) | Dose Range / Concentration & Duration | Controls Used (Positive/Negative) | Key Pharmacological Mechanisms | Critical Assessment & Limitations | Ref. |
| --- | --- | --- | --- | --- | --- | --- | --- |
| Pueraria montana var. lobata (Willd.) Maesen & S.M.Almeida ex Sanjappa & Predeep (Fabaceae) | Puerarin | *In vivo*: 3% DSS-induced colitis in male BALB/c mice. | Puerarin（purity >98 %，MP Biomedicals） 10, 50 mg/kg/day, p.o., for 7 days (co-administered with DSS) | Positive: 5-ASA (50 mg/kg/day); Model: DSS-only; Negative: Normal vehicle | ↓ NF-κB pathway (↓ COX-2, iNOS, TNF-α, IL-1β, IL-6); ↑ Nrf2/HO-1/NQO1 antioxidant signaling; ↑ Intestinal barrier integrity (restored ZO-1, occludin, claudin-1). | Lacks in vitro target validation (e.g., gene knockouts/inhibitors); prophylactic design limits therapeutic assessment; single-sex (male) bias. | Jeon et al., 2020 |
|  |  | *In vivo*: LPS-induced and Unilateral Ureteral Obstruction (UUO)-induced acute kidney injury (AKI) mouse models; *In vitro*: LPS-stimulated Raw264.7 macrophages. | *In vivo*: 50, 100 mg/kg/day, i.p. (prophylactic design, 3 days prior to LPS); *In vitro*: 50 μM pre-treatment for 12 hours. | LPS-only / UUO-only; Negative: Vehicle; Mechanistic Controls: SRT1460 (FoxO1 activator) & Anisomycin (JNK activator) used for pathway validation (Lacks clinical positive control) | ↓ M1 macrophage polarization; Directly binds to the TIR domain of MyD88 to block TLR4/MyD88 interaction; ↓ downstream NF-κB p65 and JNK/FoxO1 signaling axes | Mechanistically informative direct MyD88 binding/rescue experiments. Limitation: AKI rather than intestinal model; lacks primary human macrophage validation. | Hu et al., 2024b |
|  |  | *In vivo*: 3% DSS-induced colitis in male C57BL/6J mice; macrophage depletion/adoptive transfer; TCRδ -/- knockout mice. *In vitro*: LPS- or Amuc-protein-stimulated RAW264.7 cells. | Puerarin (Purity ≥98%, HPLC, Sigma-Aldrich) *In vivo*: 120, 160 mg/kg/day, i.p., for 7 days (co-administered with 3% DSS). *In vitro*: 10 μM, 20 μM for 24 hours (LPS or Amuc-protein challenge). Minimal active concentration tested: 10 μM. | Positive: Mesalazine (200 mg/kg/day, p.o.) Negative: Normal control + PBS Mechanistic Controls: TCRδ -/- mice, Clodronate liposome depletion group. | ↓ M1 macrophages/neutrophils and TLR2/NF-κB signaling; ↑ M2-like repair (mTOR, MHC I/II); inhibition of A. muciniphila/Amuc_2172-driven M1 activation; modulated Th17/TCRγδ axis. | Macrophage/TCRδ-focused validation. Limitations: prophylactic concurrent dosing, male-only model, high i.p. doses with limited oral translatability. | Tao et al., 2024 |
|  |  | *In vivo*: DSS-induced colitis in male C57BL/6J mice. *In silico*: Network pharmacology and molecular docking (Targeting TNF-α, IL-1β, IL-10). | Puerarin (Purity and source not specified) *In vivo*: 200 mg/kg/day, p.o. (gavage), for 2 weeks (Therapeutic design: administered after 1-week DSS induction). *In vitro*: No in vitro cellular models were conducted. | Positive: Sulfasalazine (SASP, 200 mg/kg/day, p.o.) Disease Model: DSS + Normal saline (p.o.) Negative: Normal control + Normal saline | ↓ IL-1β, IL-6 and TNF-α; reshaped microbiota (↓ Alistipes, P. copri, Veillonella; ↑ Desulfovibrionaceae.); normalized tryptophan and purine metabolites (3-HAA, guanosine). | Therapeutic post-induction design with Sulfasalazine and multi-omics. Limitations: no puerarin source/purity reporting; *in silico* targets not validated in vitro. | Zou et al., 2023 |
|  |  | *In viv*o: TNBS (100 mg/kg)-induced ulcerative colitis in female Sprague-Dawley rats. In vitro: None. | Puerarin (Sigma-Aldrich; Purity not reported in the provided text) *In vivo*: 200 mg/kg/day, p.o. (gavaged), for 14 days. | Positive control: None.  Disease Model: TNBS + normal saline (p.o.) Negative control: Normal untreated rats + normal saline (p.o.) | ↑ Goblet cell differentiation & mucin secretion (restores mucus barrier). Modulates microbiota (↓ luminal mucin-utilizing bacteria) & ↑ SCFAs. | Distinct mucus barrier/SCFA focus. Limitations: no positive control, dose-response or *in vitro* validation; purity not reported; female bias. | Wu et al., 2020 |
|  | Daidzein | *In vivo*: High-fat high-sucrose (HFHS) diet-induced obesity in male C57BL/6J mice.  *In vitro*: 3T3-L1 preadipocytes; HEK293T cells. | Daidzein (Cayman Chemical; no purity) *In vivo*: 1.0 g/kg chow (mixed in diet), for 12 weeks. *In vitro*: 25 μM (luciferase assay). | Positive control: Rosiglitazone (only used in vitro). Disease Model: HFHS diet-fed mice. Negative control: DMSO (in vitro). | PPARγ activation; ↑ adiponectin; ↓ MCP-1/TNF-α, adipocyte hypertrophy, CD11c+ macrophage infiltration; improved insulin sensitivity. | Relevant PPARγ validation. Limitations: obesity rather than IBD model, no *in vivo* positive control, purity not reported, male-only model. | Sakamoto et al., 2014 |
|  |  | *In vivo*: Cisplatin-induced nephrotoxicity (kidney injury) in male C57BL/6 mice.  *In vitro*: HK-2 cells (human kidney epithelial cells). | Daidzein (Purity > 98%, Nanjing Zelang Medical Technology Co. Ltd.) *In vivo*: 200 mg/kg/day, i.p., for 2 days. *In vitro*: 30 μM for 24 h. | Positive control: None. Disease Model: Cisplatin (25mg/kg, i.p.) + 5% DMSO/saline vehicle. Negative control: Vehicle alone (also tested daidzein alone). | ↓ Renal cell apoptosis.  ↓ Oxidative/nitrative stress (↓ NOX-2, HNE, nitrotyrosine) &↑ GPX, SOD, GSH). ↓ Inflammation (↓ CD11b+ macrophages, TNFα, IL-10, IL-18, MCP-1). | Purity reported and utilizes a therapeutic/rescue dosing timeline. Limitations: model divergence；kidney injury not intestinal inflammation; no positive control; male-only model. | Meng et al., 2017 |
|  |  | *In vitro*: LPS (100 ng/ml)-stimulated primary hepatocytes (isolated from male C57BL/6 mice). *In vivo*: None. | Daidzein (MCE, USA; Purity not reported in the text) *In vitro*: 100 μM (typically for 6h to 24h, matching LPS stimulation). *In vivo*: Not applicable. | Positive control: None.  Disease Model: LPS + 0.1% DMSO vehicle. Negative control: Normal cells + 0.1% DMSO vehicle. | ↓ AST/ALT, inflammatory cytokines and ROS; inhibited ERK1/2/NF-κB signaling; ↑ SOD activity and Nrf2, ↓ Keap1. | Strengths: Detailed mechanistic mapping (MAPK/NF-κB and Keap1/Nrf2) using primary cells. Limitations: liver rather than gut model, in vitro only, purity not reported, male-derived cells. | Yu et al., 2020 |
|  |  | *In vivo*: Soybean meal-induced enteropathy (SBMIE) in juvenile turbot (Scophthalmus maximus L.)  *In vitro*: None. | Daidzein (Purity and source not reported in the text) *In vivo*: 40 mg/kg daidzein (supplemented in diet), fed twice daily for 12 weeks. | Positive control: None. Disease Model: Soybean meal diet (SBM). Negative control: Normal fish meal diet (FM). | ↓TNF-α and MAPK/NF-κB signaling. ↓ Oxidative stress (↓ Nrf2, HO-1, Prdx-6, NQO) & apoptosis  ↑ claudin-3, claudin-4, JAM-1, ZO-1. remodeled microbiota. | Long-term barrier/microbiome assessment. Limitations: teleost model limits mammalian IBD extrapolation; no positive control; no purity/source or *in vitro* validation. | Yu et al., 2021a |
|  |  | *In vivo*: 3.5% DSS-induced acute colitis in male BALB/c mice. *In vitro*: LPS-stimulated RAW 264.7 macrophages. | Daidzein (Sigma-Aldrich; Purity not reported in the text) *In vivo*: 10 mg/kg/day, p.o. (orally), for 7 days (concurrent with DSS). *In vitro*: 0–200 μM for 24 h. | Positive control: None. Disease Model: DSS alone (in vivo) / LPS alone (in vitro). Negative control: Normal drinking water (in vivo) / untreated cells. | ↓ MPO activity,TNF-α, IL-1β, IL-6,NO and PGE2. ↓ NF-κB signaling pathway (↓ p-IKK, p-IκB-α, and nuclear translocation of p65-NF-κB). | In vivo/in vitro macrophage-linked evidence. Limitations: single in vivo dose, concurrent prophylactic dosing, no positive control, purity not reported, male-only model. | Shen et al., 2019b |
|  |  | In vivo: Angiotensin II-induced abdominal aortic aneurysm (AAA) in male BALB/C mice. (Note: Cardiovascular model, not colitis) In vitro: None. | Daidzein (Sigma-Aldrich; Purity not reported in the text) In vivo: 0.2 mg/kg/day, i.p. (intraperitoneal injection), for 4 weeks. | Positive control: None. Disease Model: Angiotensin II (1,000 ng/kg/min, s.c.) + saline vehicle (i.p.). Negative control: Normal saline (s.c.) + saline vehicle (i.p.). | ↓ serum TNF-α, IL-1β. ↓ NF-κB and p38 MAPK signaling pathways. ↓ COX-2, MMP-2, TIMP-1, TGF-β1 gene expression; ↓ iNOS protein. | Chronic inflammation/remodeling data relevant to fibrosis. Limitations: very low i.p. dose without dose-response, no positive control, male-only model. | Liu et al., 2016 |
|  |  | *In vivo*: None. *In vitro* / Abiotic: Cell-free liposomal membranes (soybean phosphatidylcholine, PC) and aqueous chemical solutions. | Daidzein (>98%, Huike Plant Exploitation, Inc., Shanxi, China) *In vitro* / Abiotic: Isoflavone/PC molar ratio of 3% (liposome assays); 1.25× 10-5 M (radical scavenging kinetic assays). | Positive/Negative: None (Not applicable for cell-free chemical assays). Unmodified daidzein was compared directly against its artificially methylated derivatives (7-Me-D, 4′-Me-D, etc.). | Scavenging of ABTS•+, AAPH and AMVN radicals; identified 4′-OH as the key antioxidant group; slowed lipid peroxidation and reduced membrane radical diffusion. | Well-characterized biophysical/electrochemical antioxidant evidence. Limitation: abiotic system lacks cells, immunity and disease context. | Liang et al., 2008 |
|  |  | *In vitro*: Rat hepatoma H4IIE cells & Human hepatoma HepG2 cells. *In vivo*: None. | Daidzein (Purity and source not reported in the text; dissolved in <0.7% DMSO) *In vitro*: 50 to 300 μM for 24 h (Prior to 2 mM H2O2 challenge). | Positive control: None. Disease/Stress Model: 2 mM H2O2 (Oxidative stress). Negative control: Vehicle (DMSO) alone. | Mild pro-oxidant behavior; no protection from H2O2-induced MDA; activation of catalase promoter/mRNA and slightly ↑ GPx, with minimal SOD effects. | Shows antioxidant-enzyme transcriptional regulation. Limitations: high non-physiological concentrations, hepatoma lines, paradoxical pro-oxidant effect without *in vivo* validation. | Röhrdanz et al., 2002 |
|  |  | *In vitro* (Cell-free): DPPH and TEAC radical scavenging assays. *In vitro* (Cellular): H4IIE rat hepatoma cells. In vivo: None. | Daidzein; its microbial metabolites 3′-OH-daidzein and 6-OH-daidzein (Analytical grade, FLUKA). Cell-free assays: ≤50 μM. Cell culture: 100–300 μM | Positive control: Trolox (Synthetic Vitamin E, used in cell-free assays only). Disease Model: None (baseline physiological state). Negative control: DMSO vehicle (max 0.5%). | Parent daidzein showed limited antioxidant activity, whereas microbial metabolites showed greater radical-scavenging activity; parent compound induced catalase transcription. | Suggests microbiota-dependent bioactivation. Limitations: supra-physiological cellular doses, hepatoma rather than intestinal cells, no *in vivo* metabolite validation. | Kampkötter et al., 2008 |
|  |  | *In vitro* (Abiotic/Cell-free): Egg phosphatidylcholine (PC) liposomes (lipid bilayers). *In vivo*: None. | Daidzein (LC Laboratories, USA; no purity) Membrane cosolubilization: 2, 5, 10, and 15 mol% of daidzein. Antioxidant assay: 5, 50, and 200 μM(incubated at 50 °C). | Positive control: None. Stress Model: AAPH (a carbon-centered free radical generator) used to induce lipid peroxidation. Negative control: Baseline liposomes without daidzein. | Membrane localization (~15% embedded at lipid-water interface). ↓ Membrane fluidity (physically impedes lipid peroxidation). Monoanionic form (7-OH deprotonated) drives primary antioxidant efficacy. | Detailed fluorescence-based membrane data. Limitations: abiotic model, no physiological complexity, non-physiological assay temperature. | Dwiecki et al., 2009 |
| Scutellaria baicalensis Georgi (Lamiaceae) | Baicalein | *In vivo*: Ovalbumin (OVA)-induced food allergy in female BALB/c mice.  *In vitro*: Primary naive CD4+ T cells (murine); Caco-2 human intestinal epithelial cells. | Baicalein (Purity and source not reported in the provided text) *In vivo*: 20 mg/kg/day, p.o. (orally), for 12 days (administered during the OVA challenge phase). *In vitro*: 5–10 μmol/L for 48 h (T cell differentiation assays). | Positive control: Dexamethasone. Disease Model: Sham group (OVA-sensitized and challenged). Negative control: Naive mice / untreated cells. | Suggested AhR-dependent CD4+Foxp3+ Treg differentiation;↓ IgE/effector T cells; ↑ TEER and claudin-3/-4/-11/-15 expression under epithelial stress. | Immune-barrier validation and positive control. Limitations: food allergy not IBD model, female-only mice, purity not reported. | Bae et al., 2016 |
|  | Baicalin | *In vivo*: 4% DSS-induced colitis in male C57BL/6 mice. *In vitro*: LPS (1 μg/ml)-stimulated murine peritoneal macrophages. | Baicalin (98% purity, Sigma-Aldrich)  *In vivo*: 25, 50, 100 mg/kg/day, i.g. (intragastric), for 10 days. *In vitro*: LPS alone (1 μg/ml) or LPS containing the indicated concentrations (6.25, 12.5, 25, or 50 μM) of baicalin for 24–48 h. | Positive control: None. Disease Model: DSS + vehicle (in vivo) / LPS alone (in vitro). Negative control: Vehicle alone (in vivo) / untreated cells (in vitro). | Drives M1-to-M2 macrophage polarization (↑ IL-10, Arg-1; ↓ iNOS+ cells, ↓ TNF-α, IL-23). Transcriptionally modulates Interferon Regulatory Factors (IRFs): ↑ IRF4 (promotes M2) and ↓ IRF5 (represses M1). | IRF4 siRNA supports causal macrophage mechanism and purity reported. Limitations: prophylactic pretreatment, no clinical positive control, male-only model. | Zhu et al., 2016 |
|  |  | *In vivo*: TNBS-induced colitis in male Sprague-Dawley (SD) rats. *In vitro*: LPS (500 ng/ml)-stimulated RAW264.7 murine macrophages. | Baicalin (Sigma-Aldrich; no purity) *In vivo*: 30, 60, 90 mg/kg/day, i.g. (intragastrically), for 4 weeks. *In vitro*: Exact baicalin concentrations not specified in the methodology text. | Positive control: None. Disease Model: TNBS + tap water (in vivo) / LPS alone (in vitro). Negative control: Saline + tap water (in vivo) / culture medium without LPS (in vitro). | ↓ Oxidative stress (↑ SOD, CAT, GSH-Px; ↓ MDA). ↓ PGE2, MPO, IL-1β, TNF-α. ↓ Apoptosis proteins (↓ Caspase-3, Caspase-9, Cyt-c).  ↓ NF-κB activation. | Strengths: Evaluates a chronic 4-week TNBS rat model targeting oxidative stress and NF-κB pathways. Limitations: Omits exact *in vitro* baicalin doses; lacks a clinical positive control; male bias. | Shen et al., 2019a |
|  |  | *In vivo*: TNBS (5%)-induced colitis in male Sprague-Dawley (SD) rats. *In vitro*: LPS (1 μg/ml)-stimulated RAW264.7 murine macrophages. | Baicalin (purity≥98% , Sigma Chemical Co.) *In vivo*: 5, 10, 20 mg/rat/day, p.o. (gastric lavage), for 15 days. *In vitro*: "Different concentrations" for 48 h | Positive control: Mesalazine. Disease Model: TNBS + water (in vivo) / LPS alone (in vitro). Negative control: Saline + water (in vivo) / vehicle/untreated (in vitro). | ↓ TLR4/NF-κB signaling pathway. ↓ TNF-α, IL-1β, IL-6, COX-2. ↓ Leukocyte recruitment & adhesion (↓ MPO activity, ↓ MCP-1, ↓ ICAM-1). | Purity and mesalazine comparator included. Limitations: non-standard mg/rat dosing hinders reproducibility; *in vitro* doses omitted; male-only model. | Cui et al., 2014 |
|  |  | *In vivo*: TNBS-induced colitis in Sprague-Dawley rats (gender unspecified). *In vitro*: None. | Baicalin (95% purity, Sigma) *In vivo*: 25, 50, 100 mg/kg, i.g. (intragastrically), once every 2 days for 14 days. | Positive control: None. Disease Model: TNBS (intrarectally) + no therapy. Negative control: 0.9% Saline (intrarectally) + no therapy. | ↓ Th17/Treg ratio.↑ ZO-1, Occludin, MUC2;↓ MDA; ↑ GSH, SOD. Modulates gut microbiota (↓ Proteobacteria, Actinobacteria; ↑ Firmicutes, Bacteroidales_S24-7). ↑ SCFAs production. | Integrates immunity–microbiota–metabolite–barrier endpoints. Limitations: 48 h dosing without PK rationale, sex not reported, no *in vitro* validation or positive control. | Zhu et al., 2020 |
|  |  | Clinical (Human): UC patients and healthy normal controls. *In vivo*: TNBS-induced ulcerative colitis in male Sprague-Dawley (SD) rats. *In vitro*: TNF-α-stimulated FHC cells. | Baicalin (Sigma-Aldrich, St. Louis, MO, USA; no purity). *In vivo*: 50 mg/kg/day, i.g. for 15 days. *In vitro*: 100, 200, 400 ng/mL (pretreated for 1 h, then co-incubated with TNF-α for 24 h). | Positive control: None. Disease Model: TNBS + 50% ethanol vehicle (in vivo); FHC + TNF-α (in vitro). Negative control: Physiological saline (in vivo); untreated cells (in vitro); healthy human tissues. | Downregulated SP1-mediated SLC6A14 transcription; ↓apoptosis, ROS, Fe²⁺, IL-6/IL-1β and ferroptosis; ↑GSH and improved histology. | Human tissue plus siRNA/overexpression/ChIP/luciferase validation. Limitations: no standard positive control, purity not reported, male-only rats. | Sun et al., 2025a |
|  | combined Baicalein/Baicalin use | *In vivo*: Composite UC model (high-sugar/fat diet + lard + alcohol + heat/humidity chamber + E. coli infection) in Sprague-Dawley rats (gender unspecified). In vitro: None. | Baicalin, Baicalein, and combination ratios YSR (4:1) & WSR (1:1) (Sinopharm Co., Ltd.; no purity) *In vivo*: 100 mg/kg/day p.o. for 7 days. | Positive control: Sulfasalazine (100 mg/kg). Disease Model: Composite UC model + normal saline. Negative control: Normal diet/conditions + normal saline. | ↓ IL-6/IL-1β/IL-17 and NF-κB/p38 MAPK. restored cAMP, cGMP, Na+/K+-ATPase. baicalin showed prolonged colonic retention, baicalein more small-intestine/lung distribution. | Comparative combination comparison with LC-MS/MS PK rationale. Limitation: purity not explicitly reported. | Liang et al., 2019 |
| Coptis chinensis Franch., Coptis deltoidea C.Y.Cheng & P.K.Hsiao, or Coptis teeta Wall. (Ranunculaceae) | Berberine | Phase I double-blind, placebo-controlled trial in human patients (Chinese) with ulcerative colitis in clinical remission (UCDAI ≤1). | Berberine (>98% pure berberine hydrochloride, 100 mg tablets, Shanghai SINE TianPing Pharmaceutical Co.) *In vivo* (Human): 900 mg/day, p.o. (300 mg, t.i.d.), for 3 months. | Positive control: None. Disease Model: Human UC patients + Placebo + Mesalamine. Negative control: Placebo. | Was associated with reduced mucosal Geboes score by 30% within berberine group; low plasma exposure (3.5 nmol/L) suggests local intestinal action; well tolerated; no significant placebo-adjusted biomarker/cytokine changes. | "First-in-human" Phase I RCT with PK/safety monitoring. Limitation: very small sample (n=12 vs. 4), limiting statistical power. | Xu et al., 2020a |
|  |  | *In vivo*: Male C57BL/6J damp-heat UC model. *In vitro*: Murine RAW264.7 macrophages stimulated by LPS (1 μg/ml). | Berberine (HPLC purity > 98%, Shanghai Yuanye BioTechnology Co., Ltd.). *In vivo*: 25, 50, 100 mg/kg BBR, p.o. for 7 days. *In vitro*: BBR 3.125, 6.25, 12.5 μM for 24 h. | Positive clinical control: Sulfasalazine. Disease Model: DSS/high-fat/high-sugar/temperature/humidity-induced UC mice; LPS-stimulated RAW264.7 cells. Negative control: Standard housing/untreated cells. | Direct binding to IRGM1 → inhibits PI3K/AKT/mTOR pathway. ↓ TNF-α, IL-6, IL-1β; ↓ MPO activity; restores tight junction proteins ZO-1, occludin, claudin-1. | *In vivo*/*in vitro* design with target identification. Limitations: no clinical validation, bioavailability not addressed, single positive control. | Meng et al., 2024 |
|  |  | Network pharmacology/docking, and molecular dynamics (Desmond). *In vivo*: 3% DSS-induced colitis in male C57BL/6 mice. *In vitro*: LPS-stimulated NCM460 cells (human normal colon mucosal epithelial cell line). | Berberine (>97% purity, Jiang Lai Biotechnology) *In vivo*: 100 mg/kg/day, p.o. (gavage), for 7 days (therapeutic regimen starting on day 4 of DSS). *In vitro*: 10 µmol/L for 24 h. | Positive control: Mesalazine (100 mg/kg/day in vivo); KC7F2 (20 µM HIF-1α inhibitor in vitro). Disease Model: DSS/LPS. Negative control: saline/normal medium. | Targeted blockade of the TLR4/NF-κB/HIF-1α signaling axis (↓ TLR4, ↓ p-NF-κB p65, ↓ HIF-1α).↓ IL-6, TNF-α, IL-1β mRNA and protein. In silico validation reveals high-affinity binding of BBR to TLR4, NF-κB, and HIF-1α pockets. | Computational and experimental support with two controls and purity reporting. Limitations: single *in vivo*/*in vitro* dose and male-only mice. | Li et al., 2024a |
|  |  | *In vivo*: 3% DSS-induced colitis in male C57BL/6 mice. *In vitro*/Co-culture: EGC–IEC–immune co-culture systems. | Berberine chloride (Sigma-Aldrich; no purity) *In vivo*: 100 mg/kg/day, p.o. (gavage), for 11 days. *In vitro*: in vitro doses not specified. | Positive control: None. Disease Model: DSS + vehicle (sterile water). Negative control: Normal + vehicle. | Restores enteric glial cells (EGCs) residence (↑ GFAP).↑ GDNF and ↓ Substance P. Restores ZO-1/E-cadherin localization, ↓ FITC-dextran permeability.↓leukocyte adhesion/chemotaxis and T-cell reactivity. | Distinct neuro–immune–epithelial mechanism with co-culture evidence. Limitations: no positive control, in vitro doses and purity not reported, male-only model. | Li et al., 2020a |
|  |  | *In vivo*: 5% DSS-induced colitis in male Sprague-Dawley (SD) rats. Includes a pseudo-germ-free (PGF) model. *In vitro*:Caco-2 cells treated with bacterial culture supernatants plus AhR siRNA. | Berberine ( 98% purity; Shanghai Boyun Biotech Co., Shanghai, China) *In vivo*: 40 mg/kg, p.o. daily for 7 days. *In vitro*: Culture supernatant (CS) diluted 1:10 for 24 h; siRNA transfection for 72 h. | Positive control: None. Disease model: DSS-induced colitis; PGF colitis. Negative control: Normal rats or normal-CS; vehicle-treated Caco-2 cells; siNC for AhR knockdown. | ↑ ZO-1/occludin;↓claudin-2/permeability;↓ TNF-α, IL-1β, IL-6; ↑ IL-10, IL-22; ↑ Lactobacillus, Clostridium, Bacteroides, Akkermansia; microbial tryptophan metabolism → AhR activation. | Multi-model design supports microbiota–AhR involvement.Limitations: Lacked a positive drug control; short-term design; human bioavailability remains unverified. | Jing et al., 2021 |
|  | Epiberberine | *In vivo*: 3% DSS-induced UC model in male C57BL/6 wild-type mice and Fxr-/- (knockout) mice (7-8 weeks old). *In vitro*: NCM460 (human normal colon epithelial), IEC-6 (rat intestinal epithelial), and HEK293T cells. | Epiberberine (Herb Substance Biotechnology Co., Ltd, Chengdu, China; 98% purity).  *In vivo*: 10, 40 mg/kg, p.o. daily for 7 days (concurrent with DSS).  *In vitro*: 5, 20 μmol/L for 24 h (in cellular inflammation/FXR inhibition models). | Mechanistic controls: Fxr-/- knockout mice (in vivo); siRNA-FXR, FXR inhibitor TUDCA (200 μmol/L), and FXR site-directed mutants (in vitro). Negative control: vehicle/untreated cells. | Activates FXR and restores bile acid homeostasis; ↓ NF-κB, IL-6, IL-1β; ↑ IL-10; direct FXR binding validated; effects reduced in Fxr−/− or FXR-silenced/inhibited models. | Relatively extensive target validation (DLR, CETSA, SIP, DARTS, IP-MS, ITC, mutagenesis). Limitations: acute DSS only, no clinical validation, FXR-focused scope. | Chen et al., 2025a |
|  | Palmatine | *In vivo*: 5% DSS-induced UC in male Sprague Dawley (SD) rats. *In vitro*: DSS-treated NCM460 cells. | Palmatine (PAL) (Purity 98%, Yuanye Biological, China). *In vivo*: 50, 100 mg/kg, p.o. daily for 7 days (concurrent with DSS). *In vitro*: 50, 100 μg/ml, co-cultured for 24 h. | Positive clinical control: Mesalazine；Ferrostatin-1 (Fer-1). Disease model: DSS-induced rats; DSS-induced NCM460 cells. Negative control: Healthy control group / untreated cells | Anti-inflammatory and anti-ferroptotic: ↓ IL-18, IL-1β, IL-6, TNF-α, MDA, NO, LDH, iron load; ↑ GSH, GPX4, phospho-Nrf2; ↓ ACSL4, COX-2, Nrf2, HO-1. | Uses Fer-1 and mesalazine with inflammation/ferroptosis readouts. Limitations: docking only for target inference, acute model, no clinical validation. | Ji et al., 2024 |
|  | Berberrubine | *In vivo*: 3% DSS-induced colitis in male Balb/c mice. *In vitro*: None. | Berberrubine (BB) (>95.58% purity via HPLC). *In vivo*: 10, 20 mg/kg/day, p.o. (gavage), for 7 consecutive days. | Positive controls: Sulfasalazine,Berberine. Disease Model: DSS + 0.5% CMC-Na vehicle. Negative control: Distilled water + 0.5% CMC-Na vehicle. | ↓ MPO activity and cytokines.↑ TJ proteins: ZO-1, ZO-2, claudin-1, occludin. ↑ mRNA expression of mucin-1 and mucin-2. ↓ Epithelial apoptosis (↓ Bax/Bcl-2 ratio; ↑ Bcl-2, ↓ Bax). | Lower-dose activity vs. parent berberine/SASP and purity reported. Limitations: no upstream target or *in vitro* validation, male-only model. | Yu et al., 2018 |
| Glycyrrhiza uralensis Fisch. ex DC., Glycyrrhiza inflata Batalin, or Glycyrrhiza glabra L. (Fabaceae) | Glycyrrhizin | *In vivo*: TNBS-induced colitis in male BALB/c mice (5–6 weeks old). *In vitro*: Primary BMDCs and BMDMs (from female BALB/c mice) co-cultured with naive CD4+ T cells isolated from spleens. | Glycyrrhizin (Tokyo Chemical Industry, Tokyo, Japan; no purity). *In vivo*: 50 mg/kg, i.p. (intraperitoneally), once every 2 days for 5 days. *In vitro*: 10, 50 μg/ml for 2-4 days. | Positive control: None. Disease Model: TNBS + PBS vehicle. Negative control: 50% EtOH (TNBS vehicle) + PBS vehicle. | Inhibition of HMGB1 release/function; shifted APCs toward regulatory DCs and away from inflammatory DC/macrophage phenotypes; suppression of Th17/Th1 cytokines without increasing Tregs. | HMGB1–APC–Th17-focused immunological evidence. Limitations: Non-translational i.p. route;no rescue design or positive control;mixed-gender source models. | Chen et al., 2017b |
|  | glycyrrhizic acid | *In vivo*: 3% DSS-induced acute colitis in female C57BL/6J mice;DiR-nanocrystal distribution. *In vitro*: LPS-RAW264.7, Caco-2 and wound assays. | Piperine (Nanjing Jingzhu Bio-Technology Co., Ltd; purity 97%)/glycyrrhizic acid (Nanjing Jingzhu Bio-Technology Co., Ltd; purity 96%) nanocrystals. *In vivo*:PIP 10 mg/kg + GA 5 mg/kg, oral, once daily for 7 days. *In vitro*: 1.25–20 μg/mL for 12–24 h. | Positive control: None. Disease model: DSS-induced mice; LPS-treated RAW 264.7 cells. Negative control: Negative and formulation controls included free drugs, NCs and DiR controls. | Colon-targeted co-delivery; anti-inflammatory (↓ IL-1β, IL-17A, MPO; ↑ IL-10, TGF-β1); barrier repair (↑ ZO-1, occludin); shifts macrophages from M1 to M2 via ↓ HIF-1α and ↑ mTOR. | Integrates formulation, uptake, distribution and DSS validation. Limitations: no standard drug control; pathway evidence mainly marker-based. | Wu et al., 2024 |
|  | Licoflavone B | *In vivo*: 1.5% DSS-induced UC model in male C57BL/6 mice (6–7 weeks old). *In vitro*: None. | Licoflavone B (Yuanye Biotech., Shanghai, China; content 90%). *In vivo*: DSS 1.5% (w/v) in drinking water for 14 days; LB 40, 80, 120 mg/kg; SASP 10 mg/kg. | Positive control: Sulfasalazine. Disease model: DSS-induced UC mice. Negative control: Normal control mice without DSS or drug. | ↓ TNF-α, IL-4, IL-6, IL-1β; ↑ IL-10.Protected barrier proteins and apoptosis; remodeled microbiota.Remodeled microbiota. MAPK inhibition: ↓ p-ERK, p-p38, p-JNK. | In vivo DSS model with SASP comparator; integrates inflammation, barrier, microbiota, and MAPK data.  Limitations: no in vitro validation or direct target evidence. | Zhang et al., 2022a |
|  | 18β-Glycyrrhetinic acid | *In vivo*: TNBS，75% ethanol-induced UC in male Sprague-Dawley rats. *In vitro*: TNBS + LPS-induced UC-like Caco-2 cell model. *In silico*: Network pharmacology screening/enrichment analysis. | 18β-Glycyrrhetinic acid (18β-GA; Yuanye Co. Ltd., Shanghai, China; purity ≥ 98%). Parent extract also studied: Glycyrrhiza uralensis extract (ULE; Solarbio, Shanghai, China; purity/content not specified). *In vivo*: ULE 50–200 mg/kg/day p.o. *In vitro*: 18β-GA 0.3–1.5 μM in vitro. | Positive control: Sulfasalazine. Disease model: TNBS/ethanol-induced UC rats; TNBS + LPS-treated Caco-2 cells. Negative control: Saline-treated rats; untreated Caco-2 cells. | Activates Wnt/β-catenin to restore tight junctions and epithelial integrity: ↑ WNT3A/β-catenin, ZO-1, Occludin; ↓ TNF-α, apoptosis, and immune-cell infiltration; effects modulated by SKL2001/IWR-1. | Rat/Caco-2 validation with Wnt agonist/inhibitor support. Limitations: no direct 18β-GA target-binding evidence, unresolved extract complexity, no clinical/CRC validation. | Peng et al., 2025 |
